# Fungal Virulence and Development Is Regulated by Alternative Pre-mRNA 3′End Processing in *Magnaporthe oryzae*

**DOI:** 10.1371/journal.ppat.1002441

**Published:** 2011-12-15

**Authors:** Marina Franceschetti, Emilio Bueno, Richard A. Wilson, Sara L. Tucker, Concepción Gómez-Mena, Grant Calder, Ane Sesma

**Affiliations:** 1 Disease & Stress Biology Department, John Innes Centre, Norwich, United Kingdom; 2 Department of Plant Pathology, University of Nebraska, Lincoln, Nebraska, United States of America; 3 Instituto de Biología Molecular y Celular de Plantas, Valencia, Spain; 4 Cell & Developmental Biology Department, John Innes Centre, Norwich, United Kingdom; University of California San Francisco, United States of America

## Abstract

RNA-binding proteins play a central role in post-transcriptional mechanisms that control gene expression. Identification of novel RNA-binding proteins in fungi is essential to unravel post-transcriptional networks and cellular processes that confer identity to the fungal kingdom. Here, we carried out the functional characterisation of the filamentous fungus-specific RNA-binding protein RBP35 required for full virulence and development in the rice blast fungus. RBP35 contains an N-terminal RNA recognition motif (RRM) and six Arg-Gly-Gly tripeptide repeats. Immunoblots identified two RBP35 protein isoforms that show a steady-state nuclear localisation and bind RNA *in vitro*. RBP35 coimmunoprecipitates *in vivo* with Cleavage Factor I (CFI) 25 kDa, a highly conserved protein involved in polyA site recognition and cleavage of pre-mRNAs. Several targets of RBP35 have been identified using transcriptomics including *14-3-3* pre-mRNA, an important integrator of environmental signals. In *Magnaporthe oryzae*, RBP35 is not essential for viability but regulates the length of 3′UTRs of transcripts with developmental and virulence-associated functions. The Δ*rbp35* mutant is affected in the TOR (target of rapamycin) signaling pathway showing significant changes in nitrogen metabolism and protein secretion. The lack of clear RBP35 orthologues in yeast, plants and animals indicates that RBP35 is a novel auxiliary protein of the polyadenylation machinery of filamentous fungi. Our data demonstrate that RBP35 is the fungal equivalent of metazoan CFI 68 kDa and suggest the existence of 3′end processing mechanisms exclusive to the fungal kingdom.

## Author Summary

The rice blast fungus *Magnaporthe oryzae* is one of the most damaging diseases of cultivated rice worldwide and an emerging disease on wheat, impacting on global food security. We identify a *M. oryzae* virulence-deficient mutant defective in the production of a RNA-binding protein (called RBP35). Clear orthologues of RBP35 are absent in yeast, plants and metazoans. We find two RBP35 protein isoforms that localise in the nucleus and bind RNA. Notably, we demonstrate that RBP35 interacts *in vivo* with a highly conserved protein component of the eukaryotic polyadenylation machinery. We show that RBP35 present different diffusional properties in nuclei of distinct fungal structures, and consequently different protein/nucleic acid interactions. Further, we find that RBP35 regulates the length of 3′UTRs of transcripts with developmental and virulence-associated functions. We prove that the Δ*rbp35* mutant is affected in the TOR (target of rapamycin) signaling pathway showing significant changes in nitrogen metabolism and protein secretion. Nothing it is known about pre-mRNA 3′ end processing in filamentous fungi and our study suggest that their polyadenylation machinery differs from yeast and higher organisms. This study can provide new insights into the evolution of the pre-mRNA maturation and the regulation of gene expression in eukaryotes.

## Introduction

The rice blast fungus *Magnaporthe oryzae* causes significant economic yield losses in rice and wheat [1]–[3]. In order to develop durable and environmentally friendly control methods, it is important to expand our knowledge on the molecular mechanisms underpinning *M. oryzae*-rice interaction. On leaves, early infection is initiated by the adhesion of three-celled conidia to the surface and the development within a few hours of a short germ tube that differentiates into a penetration structure known as an appressorium. Subsequently, a hyphal peg produced in the base of the appressorium breaches the leaf cuticle and an invasive hypha (IH) then initiates the colonisation of epidermal cells. This IH is coated by a plant-derived layer called extra-invasive hyphal membrane [4], and fungal effectors which facilitate infection and/or induce host immune responses are transferred to the plant cytoplasm across this membrane [5], [6]. The fungus also secretes several fungal toxins although their definitive role in plant infection remains unclear [7]. Fungal metabolism and autophagy play a pivotal role in the establishment of blast disease [8]–[10]. Under laboratory conditions *M. oryzae* also infects roots by developing penetration structures on underground tissues such as hyphopodia [11] and pre-IH [12]. Although silencing pathways and fungal-specific small RNAs have been identified in *M. oryzae* [13]–[15], very little is known of the post-transcriptional regulatory network that control *M. oryzae* infection ability.

Eukaryotic messenger RNA (mRNA) maturation occurs through several interdependent and co-transcriptionally regulated steps that involve pre-mRNA formation, 5′end capping, splicing, 3′end polyadenylation and degradation [16]. The 3′end formation of pre-mRNAs is a two step process essential for eukaryotic gene expression [17]. First, nearly all pre-mRNAs (with the exception of some metazoan histone genes) are cleaved at their 3′end. This step involves specific endonucleolytic cleavage at a canonical site determined by polyadenylation factors (CPSF-73 in higher eukaryotes or Cft2/Ydh1 in yeast) [18]. The second step involves the polymerization of the adenosine tail by poly(A) polymerases. Poly(A) tail length varies depending on the organism (∼200 residues in higher eukaryotes and ∼70 in yeast) and influence mRNA stability, translation, and transport. Isoforms of mRNAs with different exon content or 3′UTR lengths can be generated by alternative (or non canonical) polyadenylation, a mechanism that regulates the presence of cis elements in the mRNA. Proteins involved in alternative polyadenylation include Cleavage Factor I in metazoans (CFI_m_) and Hrp1 in yeast [19], [20]. The cis elements present in the 3′UTRs including microRNA target sites modulate gene expression by affecting cytoplasmic polyadenylation, subcellular localization, stability, translation and/or decay of the mRNA [21], [22]. In *Caenorhabditis elegans*, 43% of the genes analyzed contain more than one 3′UTR isoform [23]. In humans, about a quarter of 3′UTRs analysed also contain two or more polyadenylation signals [24].

RNA-binding proteins play a major role during all steps of RNA metabolism. They associate with RNAs and proteins to form ribonucleoprotein complexes (RNPs). The functional diversity of RNPs depends on the constituent RNA-binding proteins, which possess the dual ability to recognise cis-acting elements present in their RNA targets (primary sequence and/or secondary structures) and to interact with other proteins [25]. Several types of RNA-binding domains have been identified in eukaryotes [26]. The RNA-binding domains can be present in single or multiple copies and associate with other RNA-binding domains or additional motifs which can also bind RNA directly, facilitate RNA recognition, mediate protein-protein interactions and/or regulate subcellular localisation. Consequently, the modular structure of RNA-binding proteins regulates their wide functional repertoire and their RNA-binding ability.

The RNA recognition motif (RRM) is one of the most common and ancient protein modules found in all life kingdoms including prokaryotes and viruses [27]. RRM-containing proteins participate in nearly all known events of RNA-mediated processes. The RRM contains about 90 amino acids displayed in a conserved protein fold where at least one of the two motifs (RNP-1 and RNP-2) recognises specific RNA sequences. RNA-mediated recognition by RRMs is complex and often involves not only protein-RNA but RNA-RNA and protein-protein interactions [28]. Some non-canonical RRMs interact exclusively with proteins [29], and certain RRMs bind single stranded telomeric DNA; double stranded DNA; or interact with chromatin [27], [30]. Proteins with single or multiple copies (up to five) of RRM domains have been described, and normally are found in association with additional motifs.

In this study, we investigated the involvement of a RRM protein (RBP35) in *M. oryzae* full disease symptom production. Using a combination of cell biology, biochemistry and transcriptomics, we show that RBP35 is a novel component of the polyadenylation machinery of *M. oryzae* required for alternative 3′end processing of transcripts associated with signaling and metabolism. Results indicate that RBP35 acts as a gene-specific polyadenylation factor, ultimately regulating developmental and infection-related processes in the rice blast fungus.

## Results

### Identification of a novel RNA-binding protein implicated in fungal plant infection

**Figure 1:**
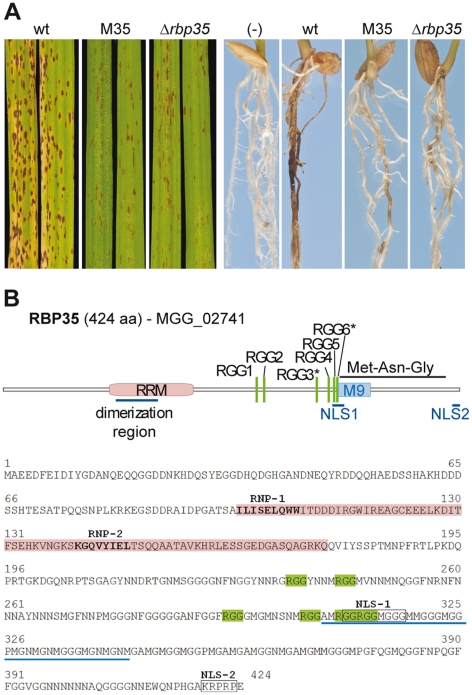
RBP35 is an RRM protein involved in fungal virulence. (**A**) *Δrbp35* strains show reduced disease symptoms on leaves and roots. wt: wild type. (**B**) Domain organisation of RBP35. The presence of a bipartite NLS and the M9-like motif suggests two alternative routes for RBP35 nucleocytoplasmic transport. RGG* can be methylated.

To identify genes required for *M. oryzae* root infection we generated and screened a random T-DNA insertional library on roots [12]. The mutant M35 produced reduced disease symptoms compared to the isogenic wild type strain Guy11 and was selected for further characterisation (Figure 1A). A tandem T-DNA insertion in M35 was located within a gene encoding a putative RNA-binding protein (*RBP35*, MGG_02741; Figure S1A available on line). The predicted protein is 424 amino acids (aa) long and contains one N-terminal RRM and six Arg-Gly-Gly tripeptide repeats (RGG; Figure 1B). Based on prediction tools, RBP35 is a non-cytoplasmic protein that contains a bipartite nuclear localisation signal (NLS).

We performed a BLASTP search against the non-redundant database at the National Center for Biotechnology Information (NCBI) and the *Saccharomyces* Genome Database (SGD) using the entire RBP35 protein as a query sequence. Uncharacterised proteins of ascomycetous filamentous fungi were the only RBP35 orthologues found at the NCBI; no significant matches were recognized at the SGD. We examined the *M. oryzae* proteome and found no paralogs of RBP35. We made an additional query exclusively using the RRM aa sequence looking for closest protein matches. Two hits were found, one at the NCBI with the *Drosophila melanogaster* cleavage factor I 68kDa (CFI_m_68; 37% identity, 59% similarity) and a second at the SGD with Nop13 (21% identity; 37% similarity), a nucleolar protein found in pre-ribosomal complexes. However, CFI_m_68 and Nop13 possess different auxiliary motifs and protein structure compared to RBP35. Consequently, we conclude that RBP35 is a filamentous fungus-specific RRM protein.

### *Δrbp35* shows alterations in development and secondary metabolite production

**Figure 2:**
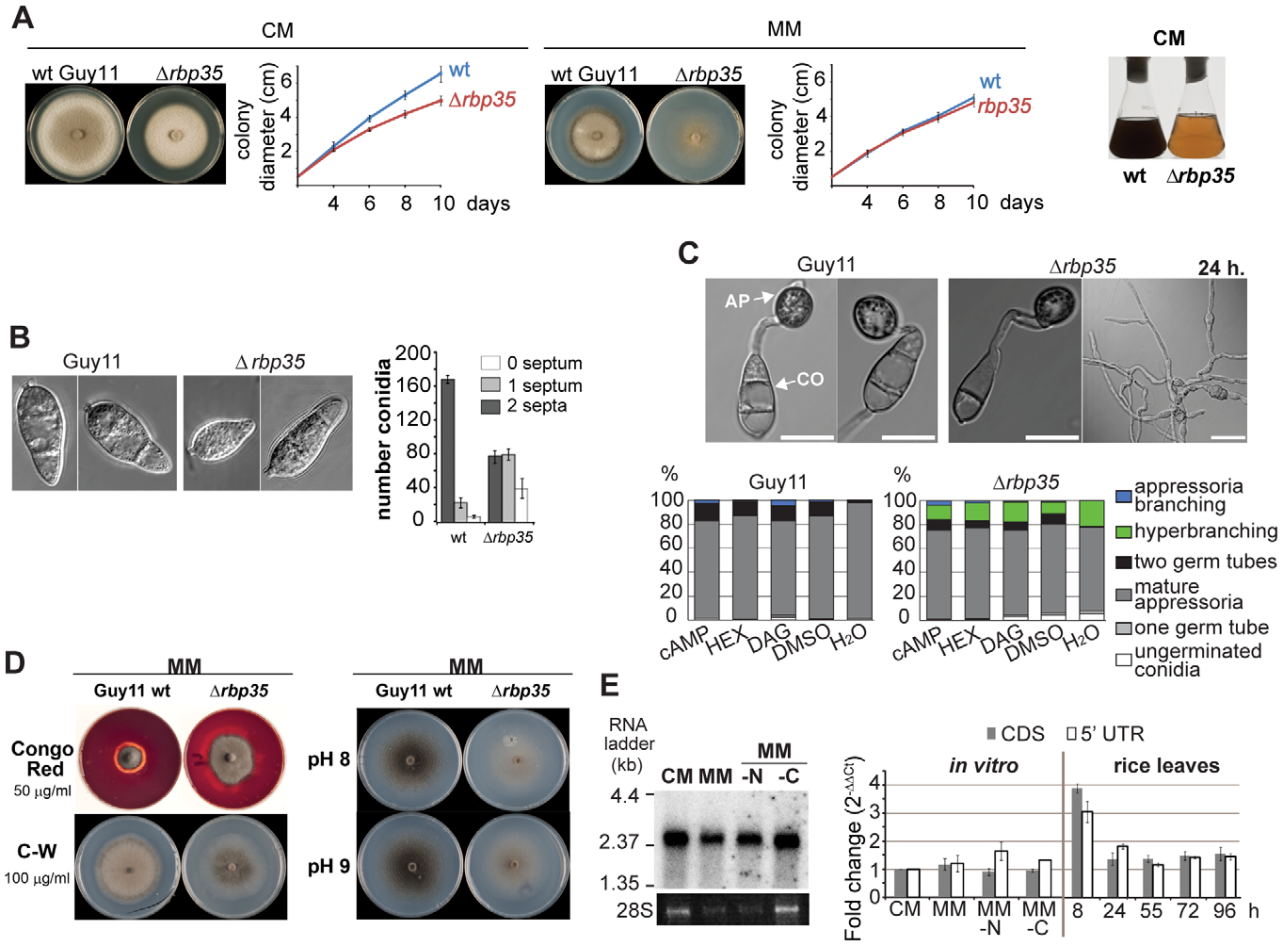
RBP35 is also implicated in *M. oryzae* development. (**A**) *Δrbp35* shows different colony morphology and growth rates (mean ± SD of three biological replica) on CM (complete medium) or MM (minimal medium). Culture filtrates of wild type and *Δrbp35* strains after 48 h on liquid CM. *Δrbp35* presents clear defects in the synthesis of pigmented metabolites. (**B**) *Δrbp35* spores show defects in conidia morphology. Scale bar: 15 µm. (**C**) Wild type and *Δrbp35* strains develop appressoria (AP) on coverslips. Conidia (CO) of *Δrbp35* produce hyperbranched hyphae at 24 h. Exogenously applied cAMP (1 mM), hexadecanodiol (HEX, cutin monomers 2 µg/mL in 1:250 DMSO) or diacylglycerol (DAG; 20 µg/ml) did not reduced hyperbranching growth in *Δrbp35*. Values represent mean percentage of three experiments. Scale bar: 15 µm. (**D**) *Δrbp35* presents cell wall anomalies (left) and withstands better alkaline conditions (right) compared to wild type strain. (**E**) Expression analysis of *RBP35* using Northern blots (left panel, ethidium bromide staining of RNA loading is shown below) and qPCRs (right panel; mean ± SD of three biological replica normalised against actin). MM-N and MM-C, minimal media minus nitrogen and carbon source respectively.

To investigate the biological function of RBP35 we generated deletion mutants of this gene and examined the mutant phenotype *in vitro* and *in planta* (Figure 1–2 and S1). *Δrbp35* strains showed fewer disease symptoms on roots and leaves as expected (Figure 1A). The mycelium of *Δrbp35* showed lower pigmentation and different morphology on both complete medium (CM) and minimal medium (MM), and reduced growth rate of *Δrbp35* was observed on CM but not on MM (Figure 2A). *Δrbp35* produced less conidia (∼50-fold) with altered morphology (septation defects) compared to the isogenic wild type strain Guy11 (Figure 2B). On plant tissues and artificial surfaces *Δrbp35* differentiated normal penetration structures (Figure 2C and S1B). However, some *Δrbp35* conidia (∼28%) produced hyperbranched hyphae at 24 h on coverslips. These defects were not overcome by addition of cAMP, cutin monomers or diacylglycerol, indicating that the mutant failed to perceive and/or respond to environmental signals. Next, we investigated the ability of the null mutant to adapt to stress-related conditions. We observed that *Δrbp35* grew faster on MM supplemented with the cell wall assembly inhibitor Congo Red and had darker mycelia in the presence of Calcofluor White compared to wild type Guy11, indicating cell wall anomalies (Figure 2D) [31]. Exposure of *Δrbp35* to alkaline pH did not alter its colony morphology whereas Guy11 showed darker mycelia, indicating the ability of *Δrbp35* to withstand high pH conditions (Figure 2D). No differential susceptibility of *Δrbp35* was observed upon exposure to oxidative, osmotic or heavy metal stresses (Figure S1C).

### The *RBP35* gene produces a single mRNA which is expressed in different media and *in planta*

We detected only one *RBP35* transcript by Northern blotting using poly(A)-enriched RNA (∼ 2.36 kb, Figure 2E). We confirmed its full length size using a combination of rapid amplification of cDNA ends (RACE) and sequencing strategies (Figure S1A). The *RBP35* gene contains an unusually large 5′ untranslated region (5′UTR) of 733 bp. Several lengths of the 3′ RACE clones were found suggesting different polyadenylation sites; 3′ RACE clones ending at 237 bp downstream of the stop codon were predominant. *RBP35* was constitutively expressed in different nutrient media and *in planta*, showing a two-fold increase in expression on leaf surfaces at 8 h after inoculation (Figure 2E).

### RBP35 binds poly(G)_30_ RNA homopolymers *in vitro*

**Figure 3:**
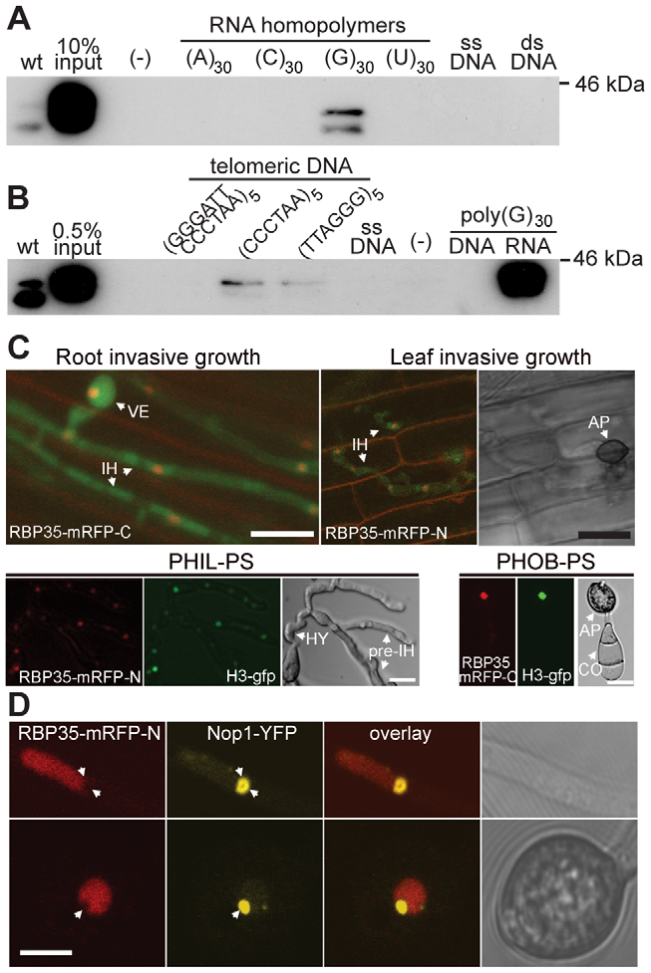
RBP35 isoforms bind poly(G) RNA homopolymers *in vitro* and show a steady-state nuclear localisation. (**A**) His-tagged RBP35 binds biotinylated poly(G)_30_ RNA homopolymers. The single- (ssDNA) and double-stranded (dsDNA) DNA derived from calf thymus was attached to cellulose beads. wt, total protein extracts from wild type. (**B**) RBP35 recognises with low affinity biotinylated single-stranded sense and anti-sense telomeric DNA repeats and not poly(dG) DNA. (**C**) Steady-state nuclear localisation of RBP35. *Δrbp35* was constructed with a cytoplasmic GFP (green fluorescent protein) to visualise its growth *in planta*. RBP35-mRFP variants colocalise in the nucleus with GFP-tagged histone H3 (H3-gfp). VE: vesicle; IH: invasive hyphae; AP: appressorium; CO: conidium; PS: polystyrene; PHIL: hydrophilic; PHOB: hydrophobic; HY: hyphopodia. Scale bar:15 µm. (**D**) RBP35-mRFP-N is excluded from the nucleolus. Scale bar: 5 µm.

**Figure 4:**
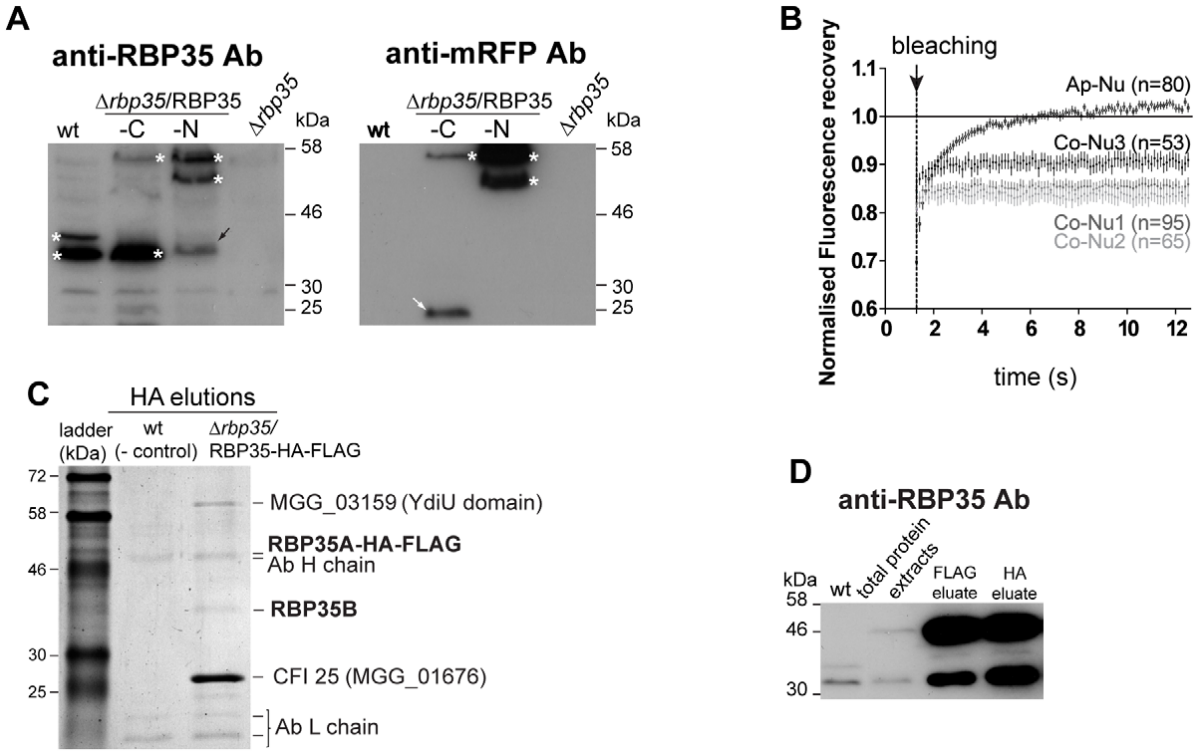
Both RBP35 isoforms showed different diffusional properties and interact with CFI25. (**A**) Two RBP35 isoforms are present in *M. oryzae*. Immunoblots of total protein extracts derived from wild type, *Δrbp35* and *Δrbp35* complemented with RBP35-mRFP variants. RBP35 isoforms (asterisks) and proteolytic products containing mRFP (white arrows) are indicated. Note the unspecific processing of RBP35-mRFP-N (black arrows). (**B**) RBP35-mRFP-N shows different fluorescence recovery kinetics in nuclei of appressoria or conidia. The relative fluorescence recovery kinetics analysed by FRAP are represented. Each data point shows the averages ± SE of analysed nuclei (n) in conidia (Co-Nu) or appressoria (Ap-Nu). (**C**) Comassie blue-stained gel of proteins that coimmunoprecipitate with RBP35-HA-FLAG after tandem affinity purification. Three proteins coimmunoprecipitate *in vivo* with RBP35A-HA-FLAG: CFI25, YdiU and RBP35B. (**D**) Immunoblots using anti-RBP35 antibodies and proteins isolated during FLAG and HA affinity purification steps. RBP35B copurifies with RBP35A-HA-FLAG indicating that both proteins interact *in vivo*.

*In vitro* binding assays were carried out using homopolymeric RNAs to determine if RBP35 is a functional RNA-binding protein (Figure 3A). For this purpose we purified His-tagged RBP35 from *E. coli* which generated two isoforms (Figure S2). This *E. coli*-purified fraction allowed us to generate polyclonal antibodies against RBP35 with good specificity (Figure 3 and 4). Results showed that RBP35 bound exclusively biotinylated poly(G)_30_ and not poly(A)_30_, poly(U)_30_, poly(T)_30_ RNA homopolymers or DNA from calf thymus (single- or double-stranded). It is well established that G-rich sequences create four-stranded structures in DNA and RNA known as G-quartets [32] and we next examined if RBP35 can recognise additional G-quadruplex structures formed by DNA sequences such as poly(dG) tracks and d(TTAGGG)_n_ repeats present in telomeres and promoter regions of ribosomal genes. RBP35 was unable to recognise biotinylated poly(dG)_30_ DNA oligonucleotides (Figure 3B). However, RBP35 recognised biotinylated single-stranded DNA oligomers containing sense and antisense telomeric repeats [d(TTAGGG)_5_ and d(CCCTAA)_5_] although its relative binding was lower compared to poly(G)_30_ RNA recognition. This result suggested that either RBP35 needed additional protein components to recognise these sequences or the RBP35 recognition was unspecific due to structural similarities.

### RBP35 shows a steady-state nuclear localisation and it is excluded from the nucleolus

To understand at what step of the RNA life cycle RBP35 participates, we generated functional RBP35-mRFP (monomeric variant of red fluorescent protein) translational fusions, which restored *Δrbp35* defects (Figure S3), to determine their subcellular localisation. Amino (RBP35-mRFP-N) and carboxy (RBP35-mRFP-C) fusions showed a steady-state nuclear localization under all developmental stages analysed *in vitro* and *in planta* (Figure 3C). Colocalisation experiments with the nucleolar protein Nop1 revealed that RBP35-mRFP was excluded from the nucleolus (Figure 3D).

### A second RBP35 isoform is generated by proteolytic processing

We identified two RBP35 protein isoforms by immunoblotting using the specific antibodies generated against RBP35 (Figure 4A): the expected full length protein (∼44 kDa; RBP35A) and a smaller variant (∼35 kDa, RBP35B). We assumed that the second protein isoform derived from post-transcriptional (i.e. alternative initiation of translation) or post-translational (i.e. proteolytic cleavage) regulation as only a single transcript of *RBP35* was detected by Northern blotting. To investigate this, we carried out western blots using total protein extracts from *Δrbp35* containing carboxy and amino RBP35-mRFP constructs (Figure 4A). Two shifted protein bands were observed using anti-RBP35 antibodies in strains expressing RBP35-mRFP-N due to the addition of mRFP. By contrast, strains expressing RBP35-mRFP-C contained only one band of high molecular weight (RBP35A-mRFP-C) that showed low affinity for the anti-RBP35 antibody, and a small band similar in size to RBP35B, which was not detected using anti-mRFP antibodies. Additionally, the presence of a low molecular weight protein band containing mRFP in strains expressing RBP35-mRFP-C corroborated that RBP35B was derived from the C-terminal proteolytic processing of RBP35A-mRFP-C.

### RBP35 showed differential kinetics in nuclei of distinct fungal structures

Fluorescence recovery after photobleaching (FRAP) is a widely used technique to measure the dynamics of RNA-binding proteins in living cells [33], [34]. To assess the ability of RBP35 to form protein complexes, the diffusional properties of RBP35-mRFP-N was monitored in the nucleus by FRAP (Figure 4B). Different kinetics of fluorescence recovery were observed after photobleaching a small region (∼0.9 µm^2^) of the nucleoplasm of appressoria or conidia. The fast recovery (less than 8 seconds) of fluorescence in the appressorium suggested that both isoforms are in a free form or associated with low molecular weight protein complexes. By contrast, the slow fluorescence recovery of RBP35-mRFP-N in the nuclei of conidia indicated binding or interactions within a large nucleic acid/protein complex. In the apical nucleus from which the first germ tube normally arises (Co-Nu3 in Figure 4B), RBP35-mRFP-N also showed smaller but significant differences in the ability to recover to the initial pre-bleaching value. These results supported that RBP35 can exhibit different protein/nucleic acid interactions in the nucleus during *M. oryzae* life cycle.

### RBP35 is a component of the *M. oryzae* polyadenylation machinery

In order to determine the molecular function by which RBP35 controls fungal virulence and development, we searched for proteins that interact with RBP35. We generated a variant of RBP35 containing the HA-FLAG tag fused to its carboxy end (Figure S4A). Immunoblots corroborated the C-terminal processing of RBP35A-HA-FLAG protein, meaning that only the full length isoform contained the HA-FLAG tag in this experiment (Figure S4B). We identified three proteins by tandem affinity purification that coimmunoprecipited with RBP35A-HA-FLAG (Figure 4C and S4C): the orthologue of the metazoan cleavage factor I 25kDa (CFI_m_25; MGG_01676); a protein containing the uncharacterized YdiU domain (MGG_03159), and RBP35B. Immunoblots using anti-RBP35 antibody against FLAG and HA elutions from *Δrbp35*/RBP35A-HA-FLAG corroborated that RBP35B interacts with RBP35A (Figure 4D).

The metazoan CFI_m_ complex is a heterotetrameric complex consisting of two small subunits of 25 kDa (CFI_m_25) and two large subunits of 59 (CFI_m_59), 68 (CFI_m_68) or 72 kDa [20]. CFI_m_25 enhances the efficiency of poly(A) site cleavage by selecting a canonical or non-canonical poly(A) site and recruiting the 3′end processing machinery [17], [35]. The recent crystal structure of a human complex CFI_m_25/CFI_m_68/RNA has established several parameters which identify likely orthologs of CFI_m_68 RRM. Remarkably, RBP35 orthologues of *Aspergillus flavus* and *Neurospora crassa* have been found among them [20]. The RRMs of the two CFI_m_68 subunits facilitate RNA looping and enhance the RNA binding by CFI_m_25 within the heterotetrameric complex. This RNA looping could explain a mechanism for a non-canonical polyA site selection and the essential involvement of CFI_m_68 in alternative polyadenylation [20]. Consequently, one role of *M. oryzae* RBP35 inferred from its structural homology with CFI_m_68 RRM and its interaction with CFI25 is its participation in canonical or alternative 3′ end processing of pre-mRNA targets.

### Transcriptome analysis led to the identification of mRNAs processed by RBP35

**Figure 5:**
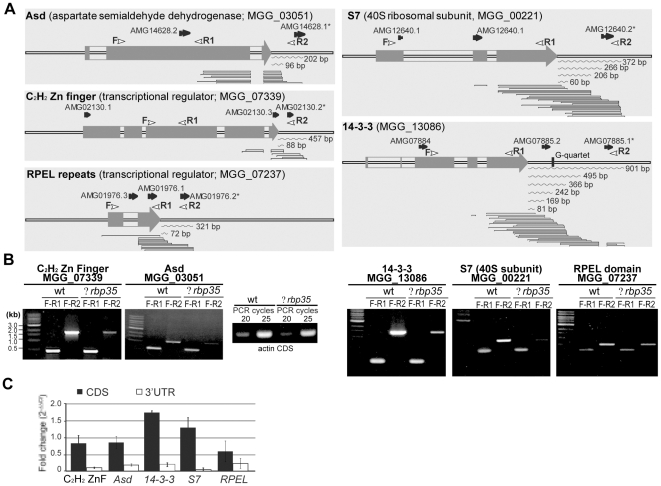
RBP35-dependent transcripts showed altered 3′UTR processing. (**A**) Analysis of 3′UTRs using genomically aligned clusters of ESTs. Microarray probes are shown in black arrows; probes marked with asterisks are down-regulated in *Δrbp35*. Curve lines represent 3′UTR lengths derived from ESTs. White arrowheads indicate location of primers used for analysis of 3′UTRs in *Δrbp35*. (**B-C**) Comparative analysis of transcript abundance of RBP35-dependent mRNAs in wt and *Δrbp35* by RT-PCR (B) and qPCR (C). cDNA concentration of wild type and *Δrbp35* was normalised using actin. Transcripts with long 3′UTRs either possess higher instability or are less abundant in *Δrbp35*.

To identify potential genes controlled by RBP35, we compared the transcriptomes of Guy11 and *Δrbp35*. Alterations in the 3′ end processing of RBP35 targets will be reflected by differential expression between microarray-oligos representing the coding sequence (CDS) or 3′UTR of the same gene. Out of the 159 genes identified in the transcriptome profiling as being differentially regulated in Δ*rbp35* compared to the wild type strain, 39 genes contained two or more oligos in the microarray chip (Figure S5). Five of these genes showed down-regulation in microarray-oligos located in their 3′UTRs and no changes in microarray-oligos located in their CDS (Figure 5A). These genes encoded a 14-3-3 protein (MGG_13086), the 40S ribosomal subunit S7 (MGG_00221), the Asd enzyme (aspartate semialdehyde dehydrogenase, MGG_03051), and two transcriptional regulators (MGG_07339 and MGG_07237). The ESTs matching these gene regions indicated their ability to produce transcripts with short and long 3′UTRs. We confirmed that their mRNAs showed altered 3′ end processing by RT-PCR and qPCR, i.e. transcripts with long 3′UTRs were less abundant in the *Δrbp35* mutant background compared to the wild type (Figure 5B and 5C). These results suggested an involvement of RBP35 in their alternative pre-mRNA 3′end processing.

### TOR pathway, protein secretion and nitrogen metabolism are affected in *Δrbp35*

**Figure 6:**
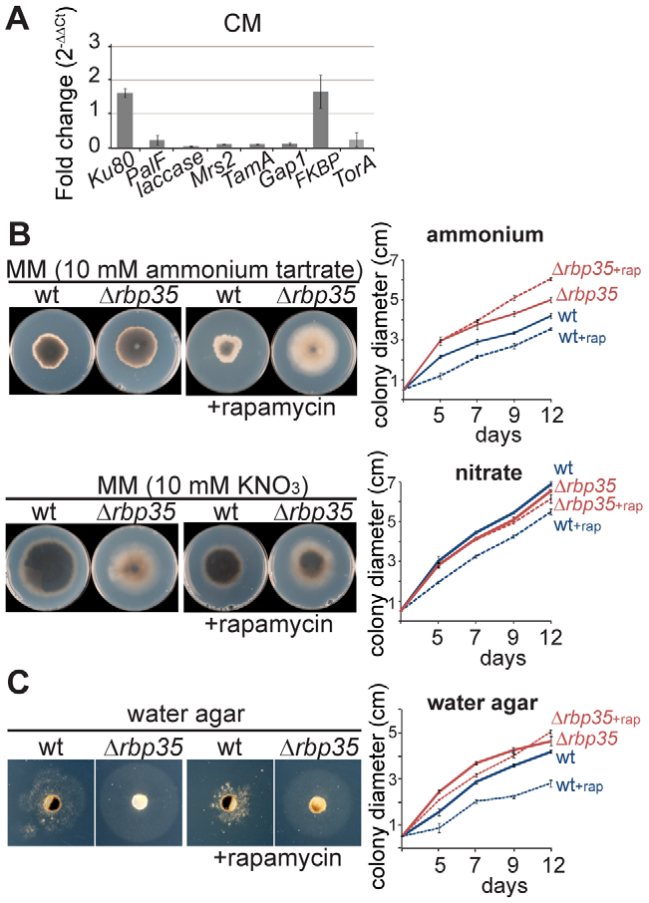
*Δrbp35* shows defects in nitrogen metabolism and TOR signaling. (**A**) Validation by qPCR of up- and down-regulated genes identified in *Δrbp35* using microarrays. Data represent means±SD (n = 3). Fungal mycelium was grown on liquid CM (complete medium). (**B**) The accelerated growth rate of Δ*rbp35* in the presence of ammonium tartrate as unique nitrogen source indicates defects in nitrogen assimilation. This effect is enhanced by inhibiting the TOR pathway with rapamycin. (**C**) Analysis of autophagy in water agar reveals that *Δrbp35* possess more efficient autophagy and is more resistant to rapamycin than the wild type strain.

We also classified the 159 genes up/down-regulated into functional groups to better understand the phenotypic defects of *Δrbp35* at the molecular level (Table S1). Eight signaling-related genes showed differential expression in *Δrbp35*. Among them, three genes encoding proteins that link extracellular stimuli to numerous signaling cascades were down-regulated, such as modulators of the small membrane-anchored G-protein RAS (MGG_04946/-7.2 fold and MGG_02933/-2.1 fold) and 14-3-3 (-3.7 fold). Two genes (Tap42 and FKBP) involved in TOR (target of rapamycin) signaling showed a two fold up-regulation suggesting alterations in this cascade. Another two genes linked to cAMP-dependent signaling pathway were up- and down-regulated respectively (CPK2/2.0 fold and ACI1/-3.3 fold). Remarkably, fifty eight genes encoding secreted or cell wall-related proteins and thirty four genes functioning in energy and intermediary or secondary metabolism showed altered expression pattern in *Δrbp35* correlating with its cell wall anomalies and pigmentation defects. In particular, seven genes involved in nitrate and ammonium assimilation were down-regulated. Genes implicated in RNA metabolism and protein synthesis (21), cytoskeleton (5) and autophagy (1) were also identified. We validated by qPCR the differential expression of genes that play significant roles in nitrogen assimilation and signaling, including the TOR kinase gene which was not originally identified due to the low signal intensity (log_2_ ∼ 6) displayed by the unique microarray-oligo representing this gene in the chip (Figure 6A).

We tested *Δrbp35* defects inferred from microarray experiments. Alterations in nitrogen assimilation correlated with the accelerated growth rate displayed by Δ*rbp35* compared to Guy11 in the presence of ammonium tartrate as unique nitrogen source on MM (Figure 6B). This Δ*rbp35* growth capacity on ammonium-media was further increased by inhibiting the TOR pathway with rapamycin (inhibitor of the TOR kinase). The down-regulation of TOR kinase expression in Δ*rbp35* could explain this tolerance towards rapamycin. No differences were observed in the presence of nitrate as nitrogen source on MM. Growth in nutrient-deficient medium forces the fungus to use autophagy for further growth. In water agar medium, an enhanced growing capacity was found in Δ*rbp35* compared to Guy11 indicating that *Δrbp35* displayed an accelerated autophagy (Figure 6C). This result was consistent with the up-regulation of *ATG24* (MGG_03638/3.6 fold) found in *Δrbp35* (Table S1). The increased growth differences between the two strains in water medium amended with rapamycin confirmed the higher tolerance of *Δrbp35* to rapamycin. The TOR signaling pathway is classically known for its role as a central regulator of growth through modulation of protein synthesis, autophagy, and proliferation in response to nutrients [36]. Based on the differential expression of genes in *Δrbp35* involved in signaling, metabolism and protein secretion together with its nutrient-dependent behaviour, accelerated autophagy and higher tolerance to rapamycin, our results suggested that the TOR pathway was significantly affected in *Δrbp35*.

## Discussion

RNA-binding proteins play a fundamental role in the control of gene expression at post-transcriptional level and are responsible for regulating essential biological activities. Here, we initiated studies in the post-transcriptional mechanisms that control *M. oryzae* infection-related processes. To this end, we characterised a RNA-binding protein required for full disease symptom production in the rice blast fungus. We found an insertional mutant M35 that showed reduced lesions on leaves and roots. The T-DNA was located in a gene encoding an RRM protein with six RGG tripeptides (RBP35). The RRM domain is widely spread in eukaryotes although only a small fraction has been studied. In humans, it is estimated that about 2% of the total proteome contain at least one RRM (497 gene products out of ∼25 000 genes in the human genome) [27]. The *M. oryzae* genome encodes 76 RRM proteins and RBP35 represents the first *M. oryzae* RRM protein investigated to date. The combination of RRM and RGG modules is found in well characterised RNA-binding proteins with highly diverse functions in human and yeast (Table S2). Orthologues of RBP35 are found only in filamentous fungi.

The *Δrbp35* mutant showed defects in development (conidiation, conidiogenesis and nutrient-dependent growth), secondary metabolism, protein secretion and cell wall biosynthesis. As a result, plants infected with *Δrbp35* exhibited lack of chlorosis on leaves and reduced lesion diameter on both leaves and roots compared to disease symptoms produced by the wild type strain. These deficiencies correlated with alterations displayed by the mutant in the TOR signaling cascade, which we identified using transcriptome profiling and confirmed by qPCR and growth tests.

The *RBP35* gene is expressed throughout all developmental stages of *M. oryzae*. We confirmed that RBP35 binds poly(G)_30_ RNA homopolymers with an assay widely used to study the binding specificity of RNA-binding proteins *in vitro* [37]. Two RBP35 protein isoforms (RBP35A and RBP35B) are found *in vivo* and colocalise in the nucleoplasm. However, it is still possible that RBP35 is present in the cell cytoplasm. The human hnRNPA1 and CFI_m_68 proteins display steady-state nuclear localization and both can carry out functions in the cytoplasm (Table S2) [38], [39].

*In vivo* coimmunoprecipation experiments revealed that RBP35A interacts with *M. oryzae* CFI25. Based on this interaction and the structural and sequence similarity of RBP35 RRM motif with CFI_m_68 RRM, we conclude that RBP35 is the functional orthologue of CFI_m_68 in filamentous fungi. Interestingly, RBP35A (∼44 kDa) also coimmunoprecipitates with RBP35B (∼35 kDa) and both isoforms are required for full complementation of *Δrbp35* defects, indicating that the *M. oryzae* CFI complex is composed at least of RBP35A, RBP35B and CFI25 subunits. Moreover, we found that a YdiU protein (MGG_03159) interacts *in vivo* with RBP35A. Further research will investigate if this protein is part of the *M. oryzae* polyadenylation machinery or interacts with RBP35 in a different protein complex.

The inferred function of RBP35 in alternative 3′ end processing of pre-mRNAs drove us to search genes with altered expression patterns in their 3′UTRs using comparative transcriptomics. Among the five genes identified as RBP35 targets, alterations in the pre-mRNA processing of *14-3-3*, *S7* and *Asd* give explanation for *Δrbp35* phenotype. The 14-3-3 proteins are involved in key cellular processes and play an important role as integrators of environmental cues through modulation of signaling cascades such as TOR [40]. Altered pre-mRNA 3′end processing could affect protein expression levels or subcellular location of the 14-3-3 mRNA, justifying the signaling-associated defects of *Δrbp35*. Recently it has been shown that the activation of TOR is mediated by its association with the ribosome in yeast and humans [41]. Therefore in addition to 14-3-3, it is possible that the *M. oryzae* 40S ribosomal subunit S7 participates as an integrating factor of ribosomal signaling and TOR activity. The defects displayed by *Δrbp35* in nitrogen metabolism can be related to the Asd protein, an enzyme implicated in the biosynthesis of aa (Lys, Met, Leu and Ile). Investigation of RBP35-mediated post-transcriptional regulation of Asd could be of significant interest to the pharmacological industry since animal cells lack this enzyme [42]. The other two RBP35-dependent mRNA encode transcriptional regulators not characterized yet in *M. oryzae*.

**Figure 7:**
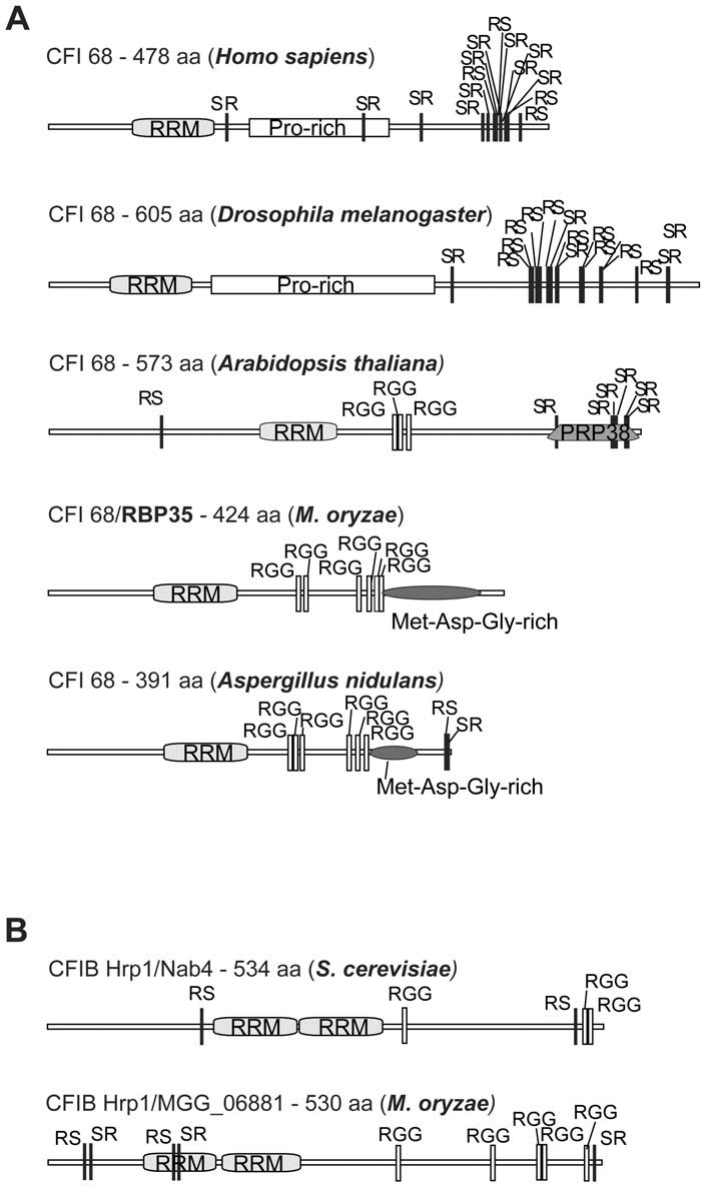
*M. oryzae* contains both yeast and metazoan CFI protein subunits. (A) Domain structure of CFI68 subunits in different kingdoms. (B) *M. oryzae* genome contains a Hrp1 homologue (MGG_06881). Hrp1 (CFIB) is the yeast equivalent of metazoan CFI_m_ complex.

In addition, the transcriptome profile analysis led us to a deeper insight into the developmental and physiological programmes deployed by RBP35. Results are consistent with RBP35 having an important role in plant virulence and fungal growth through regulation of the TOR signaling pathway. *M. oryzae* produces a range of secreted effector molecules which facilitate infection and/or induces innate and adaptive immune responses in its host [1]. More than a third of the genes (58) with expression defects in *Δrbp35* encode secreted proteins, several of which contain domains with effector functions relevant for host colonisation or modification of fungal and plant cell walls including serine proteases (3), peroxidases (2), metalloproteases (1), laccases (2), LysM effectors (1), cysteine rich proteins (1) and other enzymes (9; Table S1). RBP35 might be involved in the 3′UTR maturation of a subset of transcripts related to plant virulence. Further research will focus on the identification of RBP35-associated networks and additional direct targets and RNA binding sites using CLIP (cross-linking and immunoprecipitation) approaches [43].In addition to alternative polyadenylation of pre-mRNAs, other roles have been recently assigned to CFI_m_68 such as splicing [44], mRNA export [39], and histone 3′end processing [45]. Based on the distinct modular structure of RBP35 compared to CFI_m_68, we expect that RBP35 involvement in RNA-mediating processes will be different in *M. oryzae* (Figure 7A). Fission and budding yeast lack clear orthologues of CFI_m_25 and CFI_m_68 proteins [46]. Hrp1/Nab4 is the yeast equivalent of the CFI_m_ complex [47], and it is required for cell viability indicating its essential role in canonical and alternative polyadenylation of pre-mRNAs [19]. Obvious Hrp1 orthologues are not found in metazoans or plants [46], [48]. Intriguingly, *M. oryzae* has a clear orthologue of Hrp1 (MGG_06881, e-52; Figure 7B), which suggests that combined mechanisms regulate the 3′end processing of pre-mRNAs in filamentous fungi. This could explain the low number of genes affected in *Δrbp35* (159 genes out of the predicted 11,074 genes in *M. oryzae* genome), and corroborates that RBP35 is not essential for fungal viability but acts as a gene-specific polyadenylation factor regulating alternative 3′UTR processing of specific mRNAs.

Joint efforts and interdisciplinary approaches are necessary to identify durable control methods of blast disease in rice fields [1], [3], [49]. The identification of RBP35 as an auxiliary component of the polyadenylation machinery is an important step to unravel post-transcriptional networks that regulate *M. oryzae* plant colonisation. Future work will identify additional polyadenylation factors and cis elements present in the 3′UTRs that regulate the expression of infection-related fungal mRNAs. This area of research can lead to the identification of novel targets to control fungal diseases and will provide new insights into the evolution of the polyadenylation mechanisms in eukaryotes.

## Materials and Methods

### Fungal strains, growth conditions and plant infections

The *M. oryzae* wild-type strain Guy11 was obtained from Prof. Nick Talbot (University of Exeter, UK). Identification of DNA sequences flanking the T-DNA insertion sites, generation of targeted gene replacement mutants and growth and infection tests were performed as previously described [12].

### *M. oryzae* annotated genes

Prediction of the *M. oryzae* genes used in this study is based on the 6^th^ annotation of the genome available at The Broad Institute (http://www.broadinstitute.org/annotation/genome/magnaporthe_grisea/MultiHome.html).

### DNA cloning and purification of His-tagged RBP35 fusion protein

RBP35 protein variants were generated using multisite gateway technology (Invitrogen). The binary destination vectors pSULPH-R3R4 and pBAR-R3R4Amp which contain sulfonylurea and bialaphos resistance cassettes respectively were generated in this study. PCRs were carried out using primers detailed in Table S3 and Phusion DNA polymerase (NEB). The 5′- and 3′-RACE analysis was carried out with the SMART RACE cDNA Amplification Kit (Clontech). The *RBP35* cDNA was cloned into the expression vector pET-24 and expressed in Rosetta *E.coli* cells. His-tagged RBP35 was purified on a Ni-NTA Hitrap FF column followed by a Superdex 75 26/60 column (GE Healthcare, UK). Polyclonal antibodies against the purified protein were raised in rabbit (YORBIO, UK) and used at 1∶10000 dilution.

### RNA and DNA binding assays

*In vitro* RNA and DNA binding assay was performed using the µMACS™ Streptavidin Kit (Miltenyi Biotec, UK). Single- and double-stranded DNA-cellulose from calf thymus DNA (Sigma), byotinilated DNA oligonucleotides (TTAGGG)_5_/poly(dG)_30_ (Sigma) and biotinylated poly(A)_30_/poly(U)_30_/poly(C)_30_/poly(G)_30_ RNA homopolymers (Invitrogen) were used as bait. Binding reactions were carried out incubating 0.3 µg of RBP35 with 0.2 nmoles of bait oligo in 200 µl of binding buffer (20 mM Tris pH 8, 150 mM NaCl, 0,01% IGEPAL CA-360, 2 mM MgCl_2_, 1 mM DTT) for 1 h at 4°C. After adding 100 µl of mMACS magnetic beads the samples were applied to the column previously equilibrated with Equilibration Buffer. Washes were performed with binding buffer and protein eluted in 150 µl of binding buffer containing 1 M NaCl. Eluate aliquotes (20 µl) were loaded on 12% SDS-page gel, blotted and probed with antiRBP35 antibody.

### Protein extraction, purification, and tandem affinity immunoprecipitations

10 days-old *M. oryzae* mycelia grown on CM plates (approximately 20 cm^2^) were cut out from the agar, homogenised in a food Blender (Waring Commercial, USA) with 150 ml of liquid CM media, placed in 250 ml flasks and incubated in darkness on a shaker at 25°C/120 rpm for 2 days. Mycelia (1–3 g of wet weight) were collected by filtering through a double layer of miracloth, washed with sterile water and grounded in liquid nitrogen. Proteins were extracted from 300–400 mg of mycelia (wet weight) using 760 µl of extraction buffer (50 mM Tris pH 7.5, 5 mM EDTA, 1% Triton x-100, 10% glycerol, 2 mM Phenylmethanesulfonyl fluoride) and 20 µl/ml Protease Inhibitor Cocktail (PIC, Sigma). Cell debris were removed by centrifugation for 30 min at 4°C at 20000 g. Between 40–80 µg of total protein extract per lane were used for westerns. The following antibodies were used in western blottings: anti-FLAG (Sigma; 1∶10000); anti-mRFP (Caltag Medsystems UK; 1∶5000) and anti-HA (Sigma; 1∶10000).

### Confocal imaging and FRAP analysis

Visualisation of fungal cells and FRAP experiments were performed with a Zeiss 510 Meta confocal microscope. GFP was exciting using the 488-nm laser line from an argon ion laser, and the emission was captured using a 505 to 550 nm band-pass filter. mRFP (cherry) was exciting using the 561 nm laser line, and the emission was captured using a 575 to 615 nm band-pass filter. RBP35-mRFP-N kinetics was imaged using x63/1.4 oil objective at zoom 4. Pre- and post-bleach images were collected using 2% laser power at 561 nm and emission captured from 575 to 615 nm. The Zeiss software was set to collect ten pre-bleach images and one hundred post-bleach images; a small circular region (15 pixels diameter/0.866 µm^2^ area) was photobleached using 100% laser from 561 nm laser. Images were taken 125 ms intervals for 12.5 s when the maximal recovery of the signal was observed. Image analysis was done using the Zeiss software to measure roi (region of interest) intensities from bleached, total cell and background regions. Microsoft Excel was used to process the intensity data (background subtraction, correction for fluorescent loss and normalisation). The chart was generated using GraphPad Prism v.5.

### RNA isolation and cDNA synthesis

Infected rice leaf tissues (approx. 1 g) or *M. oryzae* mycelium were mortar-grounded in liquid nitrogen and resulting powders resuspended by vortex (30 s) in 2.5 ml of phenol and 2.5 ml of TLES buffer (100 mM Tris pH 8.0; 100 mM LiCl; 10 mM EDTA ph 8.0; 1% SDS). Subsequently, 2.5 ml of chloroform∶isoamyl alcohol 24∶1 (V/V) was added to each sample, mixed and centrifuged at 1800 g/4°C for 20 min. The same volume of 4 M LiCl was added to the supernatant and samples were gently mixed and stored overnight at 4°C. The pellet obtained after centrifugation at 12000 g was resuspended in DEPC-treated H_2_O, washed with Phenol∶Chloroform∶isoamylalcohol 24∶1 and left with 3 M NaAc and Ethanol at −80°C overnight. The resulting total RNA (20–50 µg) was treated with Turbo DNase (Ambion) and column purified using RNeasy Qiagen kit. The RNA quality was checked by automated preparation on Agilent 2100 Bioanalyzer. RNA samples (2 µg/sample) were reverse transcribed using Superscript II RT kit (Invitrogen).

### Quantitative polymerase chain reaction (qPCR)

Genes and primers are detailed in Table S3. The average threshold cycle (Ct) was normalized against actin and relative quantification of gene expression was calculated by the 2ΔΔCt method [50]. Four dilutions of all cDNA samples were used to test primer efficiency. Reactions were performed using SYBR green I kit (Roche Diagnostics). The qRT-PCR analysis was carried out using two technical repetitions from at least three independent biological experiments for each sample. Transcript levels of genes examined in *Δrbp35* are expressed as a relative value, with 1 corresponding to the transcript level in the wild type strain.

### Global gene expression profile by microarrays

Four biological replicates (each containing three technical repetitions) were independently hybridized for each transcriptomic comparison. Slides were Agilent Magnaporthe II Oligo Microarrays 4x44K (ref. 015060). Background correction and normalization of expression data were performed using LIMMA [51], [52]. LIMMA is part of Bioconductor, an R language project [53]. For local background correction the "normexp" method in LIMMA was used. The resulting log-ratios were print-tip loess normalized for each array [52]. To have similar distribution across arrays and to achieve consistency among arrays, log-ratio values were scaled using as scale estimator the median absolute value [52]. Linear model methods were used for determining differentially expressed genes. Each probe was tested for changes in expression over replicates by using an empirical Bayes moderated t-statistic [51]. To control the false discovery rate *p-values* were corrected by using the method of Benjamani and Hochberg [54]. The expected false discovery rate was controlled to be less than 5%. Hybridizations and statistical analysis were performed by the Genomics Facility at Centro Nacional de Biotecnología (Madrid, Spain).

### Tandem affinity protein purification

For purification of FLAG-HA-tagged RBP35 protein, 16 g of mycelia from liquid culture were homogenised in 30 ml of IP buffer (50 mM Tris HCl pH 7.5, 5 mM MgCl_2_, 10% glycerol, 1% Triton X-100, 1 mM PMSF, 2% PIC). Extracts were centrifuged at 20 g for 30 min, at 4°C and 25 ml of supernatant were incubated with 700 µl of anti-FLAG M2 magnetic beads (Sigma) and rinsed with TBS (50 mM Tris–HCl, pH 7.4; 150 mM NaCl, 1 mM PMSF; 2% PIC) for 2 h at 4°C with rotation. Beads were collected using a magnetic stand and washed extensively with TBS. Bound proteins were eluted by competition with 1.75 ml of FLAG peptide (150 ng/µl, Sigma), and incubated with 300 ml of HA resin (Sigma), rinsed with RIPA buffer (150 mM NaCl, 1% Igepal CA-630, 0.5% sodium deoxycholate, 0.1% SDS, 50 mM Tris HCl pH 8, 1 mM PMSF, 2% PIC) for 1.5 h at 4°C with rotation. After transferring to SigmaPrep spin columns, resins were washed with RIPA buffer and proteins eluted by incubation with 375 µl of 50 mM Tris pH 7.5, 2% SDS at 65°C for 15 min.

## Supporting Information

Figure S1 ***RBP35*** **gene deletion strategy and analysis of** ***Δrbp35*** **phenotype.**
(PDF)

Figure S2 ***E. coli*** **-derived RBP35 isoforms contain at least up to four RGG tripeptides.**
(PDF)

Figure S3 **Amino and carboxy RBP35-mRFP translational fusions are fully functional proteins.**
(PDF)

Figure S4 **RBP35A and RBP35B are part of the fungal CFI complex.**
(PDF)

Figure S5 **Identification of mRNAs with altered 3′ end processing in** ***Δrbp35.***
(PDF)

Table S1 **List of differentially expressed genes in** ***Δrbp35.***
(PDF)

Table S2 **List of characterised RRM/RGG-containing proteins.**
(PDF)

Table S3 **Primers used in this study.**
(PDF)
