# Supplementary material for: Fungal Virulence and Development Is Regulated by Alternative Pre-mRNA 3′End Processing in Magnaporthe oryzae
Source: PLoS Pathog. 2011 Dec 15;7(12):e1002441. doi: 10.1371/journal.ppat.1002441 (PMC3240610; doi:10.1371/journal.ppat.1002441)
Supplement: Figure S1 — RBP35 gene deletion strategy and analysis of Δrbp35 phenotype. (PDF) [file ppat.1002441.s001.pdf]

Supplemental Figure S1

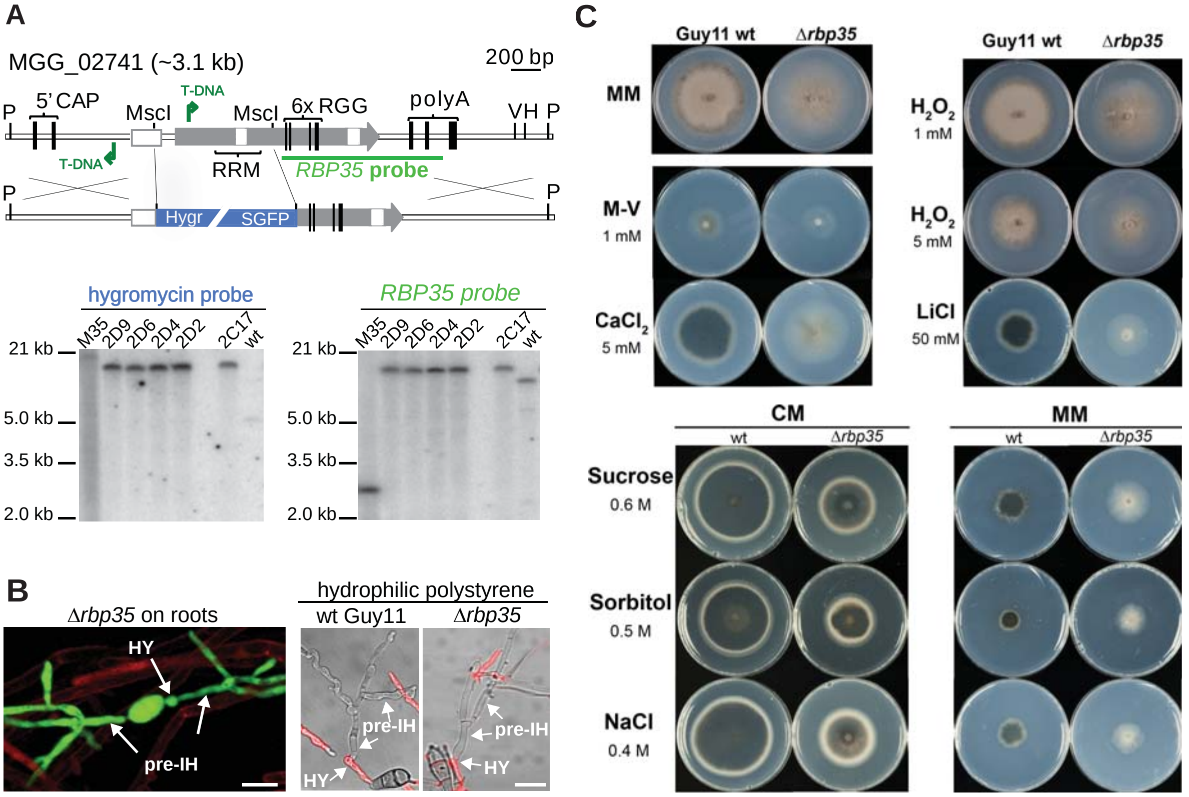

Figure S1 *RBP35* gene deletion strategy and analysis of Δ*rbp35* phenotype

(A) Targeted gene replacement strategy of *RBP35* and identification of Δ*rbp35* mutants by Shouthern blot analysis. Hybridization pattern of *Hin*dIII-digested genomic DNA revealed a single fragment of ~11.1 kb using hygromicin and *RBP35* probes in knockout mutants; an expected band of 8.8 kb using *RBP35* probe is observed in the wild type (wt) strain. RRM, RNA recognition motif; Hygr, hygromycin cassette.

(B) Δ*rbp35* develops hyphopodia (HY) and pre-invasive hyphae (pre-IH) on roots and hydrophilic polysterene surfaces as previously described in the wild type strain (Tucker et al., 2010). The red color indicates fluorescent-labeled wheat germ agglutinin which recognises chitin on the cell wall of germ tubes. Scale bar: 15 μm.

(C) Growth tests on MM or CM media supplemented with stress-related compounds. No differential susceptibility of Δ*rbp35* was observed upon exposure to oxidative stress [methyl viologen (M-V) and hydrogen peroxide (H_2O_2)], osmotic stress (sucrose, sorbitol and NaCl) or heavy metals ($CaCl_2$, LiCl).
