# Supplementary material for: Fungal Virulence and Development Is Regulated by Alternative Pre-mRNA 3′End Processing in Magnaporthe oryzae
Source: PLoS Pathog. 2011 Dec 15;7(12):e1002441. doi: 10.1371/journal.ppat.1002441 (PMC3240610; doi:10.1371/journal.ppat.1002441)
Supplement: Figure S2 — E. coli -derived RBP35 isoforms contain at least up to four RGG tripeptides. (PDF) [file ppat.1002441.s002.pdf]

Supplemental Figure S2

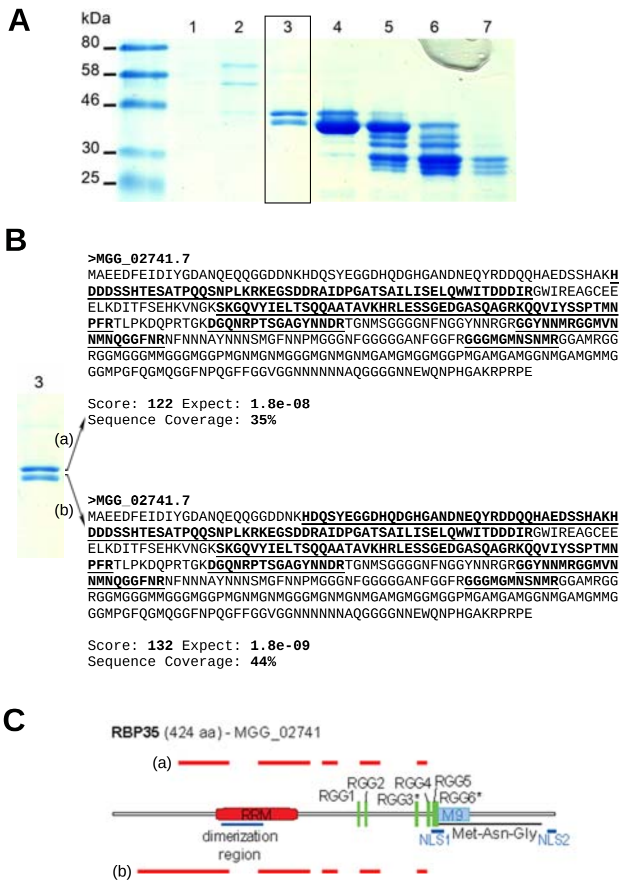

Figure S2 *E. coli*-derived RBP35 isoforms contain at least up to four RGG tripeptides

(A) Comassie blue-stained protein gel of the His-tagged RBP35 protein used in RNA-binding assays purified from *E.coli*. Lanes 1 to 7: fractions collected. Fraction 3 has been used for *in vitro* RNA and DNA binding assays.

Note the high level of premature termination of translation.

(B-C) MALDI-ToF analysis of the protein bands from fraction 3. Both the top and bottom bands have been identified as RBP35 (MGG_02741). Scores higher than 57 are significant ($p<0.05$). Unique matched peptides detected are underlined. Sequence coverage is shown in red against the protein structure (C).
