# Supplementary material for: Fungal Virulence and Development Is Regulated by Alternative Pre-mRNA 3′End Processing in Magnaporthe oryzae
Source: PLoS Pathog. 2011 Dec 15;7(12):e1002441. doi: 10.1371/journal.ppat.1002441 (PMC3240610; doi:10.1371/journal.ppat.1002441)
Supplement: Figure S3 — Amino and carboxy RBP35-mRFP translational fusions are fully functional proteins. (PDF) [file ppat.1002441.s003.pdf]

Figure S3

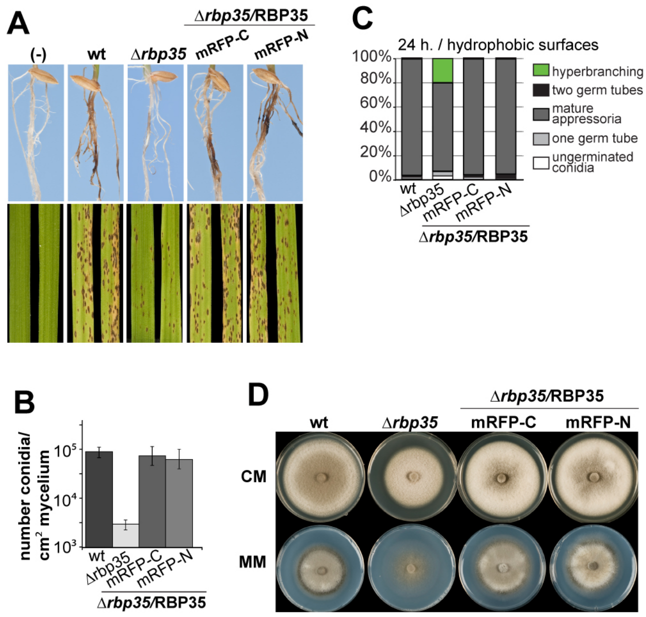

Figure S3. Amino and carboxy RBP35-mRFP translational fusions are fully functional proteins.

(A) RBP35-mRFP variants restore Δ*rbp35* virulence deficiencies on leaves and roots.

(B-C) RBP35-mRFP variants can complement Δ*rbp35* conidia production and hyperbranching defects. Values represent mean percentage of three experiments.

(D) Colony morphology on different media of wild type, Δ*rbp35* and Δ*rbp35* complemented with RBP35-mRFP variants.
