# Supplementary material for: Fungal Virulence and Development Is Regulated by Alternative Pre-mRNA 3′End Processing in Magnaporthe oryzae
Source: PLoS Pathog. 2011 Dec 15;7(12):e1002441. doi: 10.1371/journal.ppat.1002441 (PMC3240610; doi:10.1371/journal.ppat.1002441)
Supplement: Figure S4 — RBP35A and RBP35B are part of the fungal CFI complex. (PDF) [file ppat.1002441.s004.pdf]

Figure S4

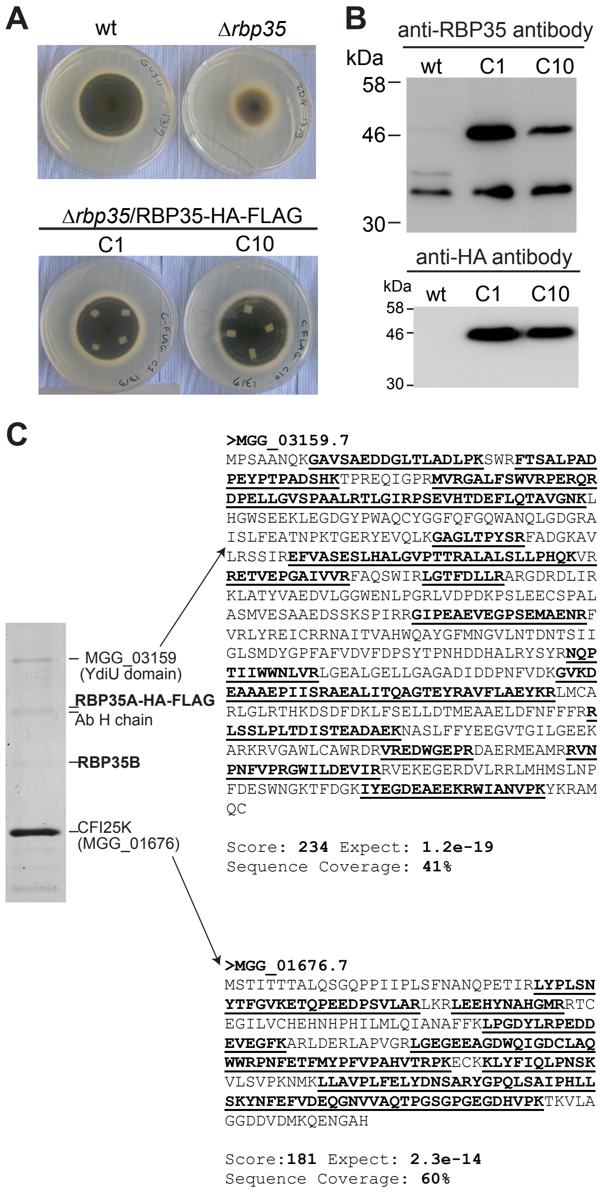

Figure S4. RBP35A and RBP35B are part of the fungal CFI complex.

(A) The C-terminal RBP35-HA-FLAG translational fusion construct restores Δ*rbp35* colony morphology on CM plates. C1 and C10 are two different Δ*rbp35* transformants containing the RBP35-HA-FLAG construct.

(B) Immunoblots of wild type (total protein extract) and Δ*rbp35*/RBP35-HA-FLAG (HA eluate) strains corroborate the C-terminal processing of RBP35-HA-FLAG.

(C) Mass spectrometry analysis of RBP35 complex identifies CFI25 and YdiU proteins. Unique matched peptides detected are underlined. Scores higher than 57 are significant ($p<0.05$).
