# Supplementary material for: Fungal Virulence and Development Is Regulated by Alternative Pre-mRNA 3′End Processing in Magnaporthe oryzae
Source: PLoS Pathog. 2011 Dec 15;7(12):e1002441. doi: 10.1371/journal.ppat.1002441 (PMC3240610; doi:10.1371/journal.ppat.1002441)
Supplement: Figure S5 — Identification of mRNAs with altered 3′ end processing in Δrbp35. (PDF) [file ppat.1002441.s005.pdf]

Figure S5

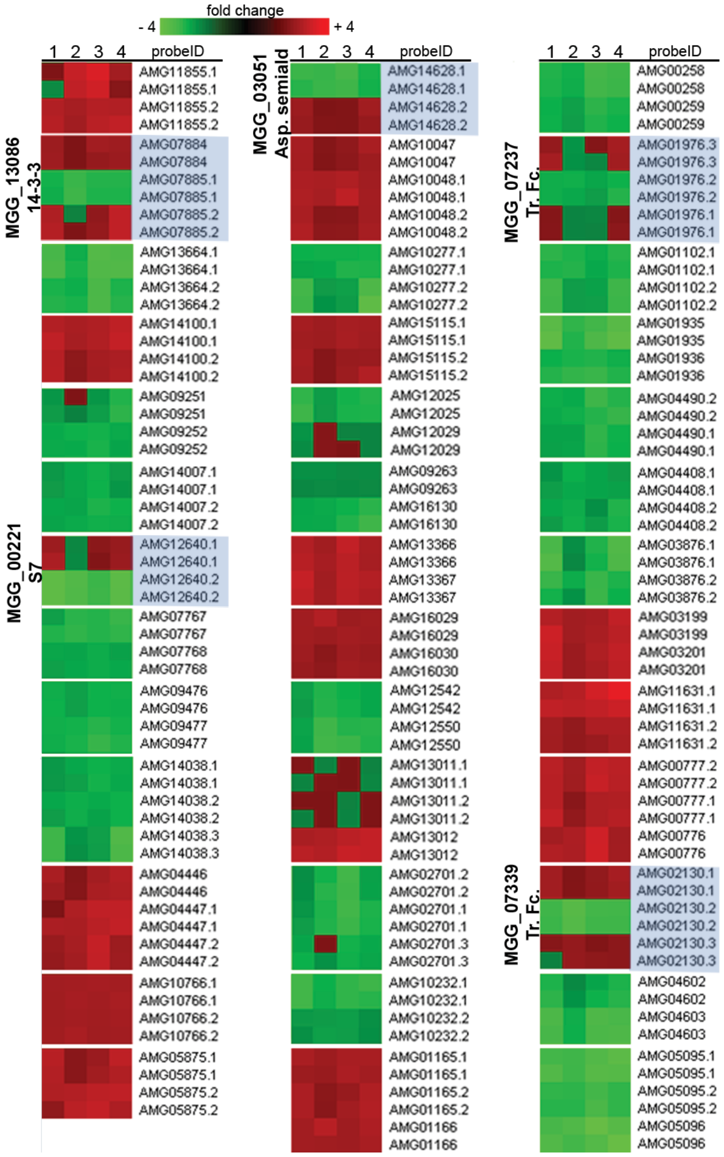

Figure S5. Identification of mRNAs with altered 3'end processing in Δ*rbp35*.

Heat map of the 39 genes up- or down-regulated in Δ*rbp35* that contain two or more microarray-oligos. Lanes 1 to 4 represent four independent hybridisations using RNA extracted from different biological samples. Five genes (indicated in blue) contain 3'UTR probes down-regulated in Δ*rbp35*.
