# Supplementary material for: Fungal Virulence and Development Is Regulated by Alternative Pre-mRNA 3′End Processing in Magnaporthe oryzae
Source: PLoS Pathog. 2011 Dec 15;7(12):e1002441. doi: 10.1371/journal.ppat.1002441 (PMC3240610; doi:10.1371/journal.ppat.1002441)
Supplement: Table S1 — List of differentially expressed genes in Δrbp35. (PDF) [file ppat.1002441.s006.pdf]

Table S1. List of differentially expressed genes in Δ*rbp35*.

[1] Using a cut-off value of two-fold change with FDR LiMMA < 0.015, 103 genes were up/down regulated (shown in bold). Additional 56 genes were found using three-fold change with FDR LiMMA <0.05. In both cases, the gene probes had at least an average log_2 signal intensity larger than 8. The five genes presenting alterations in their 3'UTRs in Δ*rbp35* are indicated in red.

[2] *S. cerevisiae* orthologues unless indicated.

[3] References for *M. oryzae* genes.

[4] Proteins listed in the fungal secretome database [1]; TM: transmembrane region; GPI: glycosylphosphatidylinositol-anchored prediction using big-PI Fungal Predictor [2].

Fold change[1]	Agilent probe	GENE ID	PROTEIN DESCRIPTION / ORTHOLOGUES[2]	REFERENCES[3]
**SIGNALING (8)**				
**-7.2**	**AMG05908**	**MGG_04946**	**7-TM; Ste14 domain (carboxyl methylation of Ras)**	
**-3.7**	**AMG07885.1**	**MGG_13086**	**14-3-3 protein**	
**-3.3**	**AMG04255**	**MGG_05531**	**ACI1 (MAC1 interacting protein 1); CFEM domain**	[3]
**-2.4**	**AMG00884**	**MGG_01615**	**arrestin / PalF (A. nidulans, response to alkaline pH)**	
**-2.1**	**AMG14431**	**MGG_02933**	**4-TM; DHHC domain (palmitoyl-transferase of Ras) / Cfa5**	
**2.1**	**AMG00778**	**MGG_01540**	**TAP42 (essential protein involved in TOR signalling)**	
**2.0**	**AMG07776.1**	**MGG_04476**	**FKBP (peptidylprolyl isomerase domain linked with TOR signalling)/Fpr3, Fpr4**	
**2.0**	**AMG14280**	**MGG_02832**	**CPK2 (cAMP-dependent protein kinase A)**	
**SECRETED/CELL WALL-RELATED[4] (58)**				
**1. CLASSICAL SECRETION PATHWAY (35)**				
**-9.0**	**AMG04581**	**MGG_05747**	**no hits**	
**-7.9**	**AMG10412**	**MGG_03755**	**SCP-like (cysteine-rich secretory protein)**	
**-7.6**	**AMG05096**	**MGG_13464**	**laccase**	
-6.7	AMG08466	MGG_09412	GPI; no hits	
-6.1	AMG13664.1	MGG_09079	GPI; no hits	
**-5.5**	**AMG05419**	**MGG_05982**	**no hits**	
-5.4	AMG14325	MGG_02863	oryzin; serine protease; GPI / Prb1 (sporulation)	
-5.0	AMG07091	MGG_08451	no hits	
-4.3	AMG06593	MGG_07790	lignin peroxidase LG6 (class II fungal peroxidase)	[4]
-4.0	AMG09836	MGG_03364	no hits	
-3.9	AMG00258	MGG_11274	tyrosinase-copper monooxygenases	
**-3.7**	**AMG10153**	**MGG_03585**	**no hits**	
-3.6	AMG06106	MGG_12942	no hits	
-3.5	AMG12550	MGG_11608	laccase (multicopper oxidase)	
-3.5	AMG07301	MGG_08589	alpha/beta hydrolase	
-3.4	AMG06881	MGG_09474	no hits	

**-3.2**	**AMG01102.1**	**MGG_01764**	**no hits**	
**-3.2**	**AMG09477**	**MGG_12773**	**metalloprotease Y (peptidase M28 family)**	
**-3.2**	**AMG13055**	**MGG_09834**	**catalase/peroxidase; involved in ROS response**	[4,5,6,7,8]
**-3.1**	**AMG15382**	**MGG_10244**	**no hits**	
-3.1	AMG03980	MGG_13019	no hits	
-3.0	AMG07152	MGG_08494	CYT P450 / Dit2 (sporulation-specific enzyme)	
**-2.8**	**AMG07849**	**MGG_04532**	**LysM (peptidoglycan binding)**	
**-2.7**	**AMG07517**	**MGG_08758**	**protease; PA (associated with vacuolar sortin receptors)**	
**-2.3**	**AMG08427**	**MGG_14950**	**no hits**	
**-2.2**	**AMG04408.1**	**MGG_05638**	**GPI; no hits**	
**-2.1**	**AMG07064**	**MGG_08436**	**subtilisin (serine-protease)**	
**-2.1**	**AMG00666**	**MGG_01466**	**WSC (carbohydrate binding)**	
**5.1**	**AMG15480**	**MGG_10394**	**no hits**	
**3.9**	**AMG13012**	**MGG_15427**	**no hits**	
3.6	AMG02403	MGG_08373	no hits	
3.5	AMG15046	MGG_08962	no hits	
**2.8**	**AMG04956**	**MGG_07609**	**no hits**	
**2.5**	**AMG00465**	**MGG_01328**	**glycosyl hydrolase family 45; endoglucanase**	
**2.0**	**AMG00051**	**MGG_10471**	**no hits**	

2. NON CLASSICAL SECRETION PATHWAY (23)

**-9.1**	**AMG12896**	**MGG_00070**	**no hits**
**-6.8**	**AMG12898**	**MGG_00071**	**no hits**
**-5.6**	**AMG15558**	**MGG_10525**	**no hits**
**-5.1**	**AMG05288**	**MGG_05893**	**no hits**
-4.4	AMG10610	MGG_12091	no hits
-4.4	AMG12494	MGG_14700	no hits
-4.4	AMG07093	MGG_08453	no hits
-4.3	AMG10858	MGG_04071	no hits
-3.9	AMG10232.1	MGG_03629	no hits
-3.6	AMG05861	MGG_04910	no hits
-3.2	AMG10998	MGG_04173	serine hydrolase
-3.2	AMG11556	MGG_00947	no hits
**-3.1**	**AMG08419**	**MGG_09381**	**DUF3328**
-3.0	AMG02881	MGG_02206	no hits
**-2.7**	**AMG14038.2**	**MGG_15025**	**no hits**
**-2.6**	**AMG00744**	**MGG_11191**	**no hits**
**-2.2**	**AMG05190**	**MGG_05826**	**epoxide hydrolase**

**-2.1**	**AMG08838**	**MGG_10035**	**no hits**	
**-2.0**	**AMG04937**	**MGG_07599**	**no hits**	
**4.0**	**AMG12941**	**MGG_00041**	**O-methyl transferase**	
3.3	AMG00777.2	MGG_11188	no hits	
**2.7**	**AMG02466**	**MGG_15364**	**no hits**	
**2.4**	**AMG02740**	**MGG_02101**	**SAM-dependent methyl transferase type 11**	
**METABOLISM (34)**				
**1. NITROGEN METABOLISM**				
-5.3	AMG04603	MGG_05759	cysteine desulfurase/Aspartate aminotransferase (molibdenum cofactor sulfurase)	
-5.1	AMG07956	MGG_13793	12-TM; nitrate transporter / NrtB (A. nidulans)	
**-4.1**	**AMG14628.1**	**MGG_03051**	**aspartate-semialdehyde dehydrogenase (arginine biosynthesis)**	
-4.0	AMG01427	MGG_08074	glutamate dehydrogenase / GdhA (A. nidulans; nitrogen catabolite repression)	
-3.2	AMG02701.1	MGG_02072	general aa permease Gap1 (modulates TOR signalling; regulated by nitrogen)	
-3.1	AMG12025	MGG_00634	nitrite reductase / NiiA (A. nidulans; nitrate assimilation)	
**-2.8**	**AMG09252**	**MGG_06492**	**transcription factor / Gal4 / TamA (A. nidulans)**	
5.2	AMG11625	MGG_00923	Serine hydroxymethyltransferase (aspartate aminotransferase superfamily)	
4.4	AMG07812	MGG_04503	subunit of carbamoyl-phosphate synthase / Cpa2 (arginine biosynthesis-urea cycle)	
**3.4**	**AMG11057**	**MGG_04210**	**ornithine acetyltransferase (arginine biosynthesis) / Arg7**	
3.1	AMG14100.1	MGG_02713	NAC domain / Egd1 (enhances DNA binding of Gal4p)	
**2.3**	**AMG04013**	**MGG_05371**	**asparagine synthetase; glutamine amidotransferase**	
**2. SECONDARY METABOLISM**				
**-10.4**	**AMG07104**	**MGG_08461**	**Zn-containing Alcohol Dehydrogenase**	
**-4.0**	**AMG01936**	**MGG_07214**	**glucosyl transferase family 25**	
-3.1	AMG15188	MGG_09945	CYT P450 / Dit2 (sporulation-specific enzyme)	
**-2.9**	**AMG10277.1**	**MGG_03662**	**phosphosulfate reductase**	
**2.5**	**AMG04981**	**MGG_07626**	**2-TM; CYT P450**	
**2.4**	**AMG02388**	**MGG_15100**	**polyketide synthase syn6**	[9,10]
**2.2**	**AMG00894**	**MGG_01620**	**Velvet / VelB (A. nidulans)**	
**3. LIPID METABOLISM**				
**-5.0**	**AMG02751**	**MGG_02110**	**2-TM; caleosin (Ca2+-binding prot in lipid-bodies; esterol synthesis)**	
-4.1	AMG00165	MGG_10170	4-TM; DUF1295 (related with sterol metabolism)	
**-2.8**	**AMG08893.1**	**MGG_06250**	**3-TM; fatty acid desaturase;esterol and sphingolipid biosynthesis**	
3.6	AMG12144	MGG_00593	carboxyl esterase	
**4. ENERGY PRODUCTION AND CONVERSION**				
**-2.2**	**AMG04366**	**MGG_05607**	**Cytochrome C heme lyase CYC3 (*S. cerevisiae*)/ CCHL (*N. crassa*)**	
**4.7**	**AMG16138**	**MGG_11021**	**ATPase (AAA family)**	
**4.3**	**AMG11631.1**	**MGG_00892**	**ATP synthase subunit C (F0 complex; proton-translocating ATPase)**	

**5. CARBOHYDRATE METABOLISM**				
3.5	AMG02386	MGG_08363	enoyl reductase / ApdC (A. nidulans)	
**2.4**	**AMG03792**	**MGG_06764**	**guanylate kinase (mannose outer chain elongation of cell wall glycoproteins)**	
**6. COENZYME METABOLISM**				
-3.6	AMG09907	MGG_03414	SDR dehydrogenases (NAD- or NADP-dependent oxidoreductases)	
**3.7**	**AMG12501**	**MGG_00312**	**NAD-dependent dehydrogenase**	
3.4	AMG08166	MGG_04738	SDR dehydrogenases (NAD- or NADP-dependent oxidoreductases)	
**2.9**	**AMG11855.2**	**MGG_00755**	**indoleamine 2,3-dioxygenase (de novo biosynthesis of NAD from tryptophan)**	
**2.1**	**AMG04742**	**MGG_07455**	**SDR dehydrogenases (NAD- or NADP-dependent oxidoreductases)**	
**2.0**	**AMG11901**	**MGG_00697**	**SDR dehydrogenases (NAD- or NADP-dependent oxidoreductases)**	
**TRANSCRIPTION REGULATION (5)**				
**-6.2**	**AMG02130.2**	**MGG_07339**	**C2H2 Zinc finger transcription factor**	[11]
**-2.7**	**AMG01976.2**	**MGG_07237**	**RPEL repeat (DNA binding repeat)**	
**4.1**	**AMG03415**	**MGG_11779**	**NFX1 Zinc finger transcription factor**	[11]
**2.6**	**AMG12974**	**MGG_00019**	**SET (histone methyl transferase) / Set2**	
**2.1**	**AMG07673**	**MGG_04401**	**JmC transcription factor**	[11]
**RNA METABOLISM, TRANSLATION, RIBOSOME BIOGENESIS (21)**				
-8.2	AMG12640.2	MGG_00221	S7 (40S ribosomal protein subunit)	
**-5.0**	**AMG07950**	**MGG_04594**	**MRS2-like (mitochondrial Mg2+-dependent RNA splicing factor)**	
-3.7	AMG03876.2	MGG_06683	3' exoribonuclease / Rrp42 (exosome complex)	
**-3.0**	**AMG12776**	**MGG_00143**	**LYAR-type C2HC zinc finger**	
4.0	AMG11474	MGG_01013	eIF3i subunit / Tif34 (essential for translation)	
**3.5**	**AMG08998**	**MGG_06321**	**tRNA synthetase class II**	
**3.4**	**AMG05875.2**	**MGG_04921**	**L23 (60S ribosomal protein)**	
3.2	AMG04447.1	MGG_05661	S8 (40S ribosomal subunit)	
**3.0**	**AMG14174**	**MGG_02762**	**DEAD-box (RNA helicase) / Dbp1**	
**2.9**	**AMG03628**	**MGG_06857**	**tRNA methylase / Trm11**	
**2.8**	**AMG10287**	**MGG_03668**	**Kap95 (karyopherin *S. cerevisiae*)**	
**2.8**	**AMG03251**	**MGG_02449**	**tyrosyl-tRNA synthetase / Tys1**	
**2.7**	**AMG10906**	**MGG_04104**	**L22 (60S ribosomal protein subunit)**	
**2.7**	**AMG04136**	**MGG_05449**	**L16 (60S ribosomal protein subunit)**	
2.6	AMG13314	MGG_09208	karyopherin / Kap104 / transportin (human)	
**2.6**	**AMG10048.1**	**MGG_13535**	**Nucleoporin / Pom152**	
**2.3**	**AMG14853.1**	**MGG_08834**	**L28 (60S ribosomal protein subunit)**	
**2.2**	**AMG15115.1**	**MGG_09894**	**S28 (40S ribosomal protein subunit)**	

**2.2**	**AMG04671**	**MGG_11426**	**Nuclear 5' to 3' single-stranded RNA exonuclease / Rat1**	
**2.1**	**AMG06297**	**MGG_05213**	**Sm-like domain / Edc3 (mRNA decapping)**	
**2.1**	**AMG16029**	**MGG_14350**	**WD repeat / Rrb1 (ribosome biogenesis-assembly protein)**	
**CYTOSKELETON/POLARITY/ADHESION (5)**				
**-3.1**	**AMG15283**	**MGG_10098**	**1-TM; Kelch repeat (which may have a cytoskeletal function)**	
**2.3**	**AMG11664**	**MGG_00874**	**Dam1 subunit (kinetochore protein of the DASH complex)**	
**2.7**	**AMG01011**	**MGG_01702**	**SH3, PX and PB1 domains / Bem1**	
**2.2**	**AMG12641**	**MGG_00222**	**dynactin p62 subunit**	
**2.0**	**AMG03817**	**MGG_14584**	**kinetochore protein (Cnl2-NKP2 family)**	
**TELOMERE BIOGENESIS AND MAINTENANCE (2)**				
3.7	AMG06435	MGG_05307	STM1 domain (telomere maintenance and mRNA stability)	
3.0	AMG00149	MGG_10157	KU80; nonhomologous end-joining (NHEJ) and telomere maintenance	
**AUTOPHAGY (1)**				
**3.6**	**AMG10249**	**MGG_03638**	**sortin-nexin; PX domain; autophagy-related protein 24**	[12]
**OTHERS (13)**				
-4.8	AMG00739	MGG_01511	12-TM; ABC transporter / Flr1	
-4.7	AMG07212	MGG_08535	3-TM; MARVEL domain (lipid-associating proteins)	
**-3.8**	**AMG07767**	**MGG_04469**	**2TM*; CYT P450**	
-3.5	AMG04541		EST near MGG_13153 (transcriptional repressor)	
-3.3	AMG11220	MGG_04329	12-TM; MFS I (Multidrug transporter-major facilitator superfamily)	
-3.1	AMG01964	MGG_07228	10-TM / Opt1 (Proton-coupled oligopeptide transporter)	
**-2.2**	**AMG08407**	**MGG_09372**	**fasciclin MoFLP1, vacuolar**	[13]
3.1	AMG13367	MGG_11754	6-TM; ABC transporter	
**2.8**	**AMG00228**	**MGG_01169**	**3-TM; epoxide hydrolase**	
**2.6**	**AMG13982**	**MGG_02633**	**8-TM; CDP-alcohol phosphatidyltransferase; phospholipid biosynthesis**	
**2.3**	**AMG10766.1**	**MGG_04002**	**RAMP4 (stabilisation of membrane proteins in response to stress)**	
**2.3**	**AMG03508.1**	**MGG_06931**	**metal-dependent glycoprotease M22; molecular chaperone**	
**2.2**	**AMG01166**	**MGG_01807**	**NEDD8conjugating enzyme/ Ubc12 (ubiquitination-proteosome degradation)**	
**UNKOWN FUNCTION (12)**				
**-10.3**	**AMG02750**	**MGG_02109**	**no hits**	
**-9.3**	**AMG04297**	**MGG_05559**	**no hits**	
**-4.1**	**AMG04314**	**MGG_05573**	**no hits**	
-3.4	AMG11634.2		EST near MGG_00884	
-3.4	AMG04490.2	MGG_05692	4-TM; no hits	
-3.2	AMG16130	MGG_12820	1-TM; no hits	

**-2.9**	**AMG05513**	**MGG_06052**	**DUF2343**
**-2.6**	**AMG14007.2**	**MGG_02654**	**2-TM; no hits**
**-2.3**	**AMG05221**	**MGG_05849**	**2-TM; no hits**
3.9	AMG09562	MGG_03176	no hits
3.2	AMG03199	MGG_11914	unknow function domain KxDL; sometimes present with RRM
**2.3**	**AMG03753**	**MGG_06739**	**no hits**

REFERENCES (Table S1)

1. Lee YH, Choi J, Park J, Kim D, Jung K, et al. (2010) Fungal Secretome Database: Integrated platform for annotation of fungal secretomes. BMC Genomics 11.
2. Eisenhaber B, Schneider G, Wildpaner M, Eisenhaber F (2004) A sensitive predictor for potential GPI lipid modification sites in fungal protein sequences and its application to genome-wide studies for *Aspergillus nidulans*, *Candida albicans*, *Neurospora crassa*, *Saccharomyces cerevisiae* and *Schizosaccharomyces pombe*. Journal of Molecular Biology 337: 243-253.
3. Kulkarni RD, Dean RA (2004) Identification of proteins that interact with two regulators of appressorium development, adenylate cyclase and cAMP-dependent protein kinase A, in the rice blast fungus *Magnaporthe grisea*. Molecular Genetics and Genomics 270: 497-508.
4. Koua D, Cerutti L, Falquet L, Sigrist CJ, Theiler Gx, et al. (2009) PeroxiBase: a database with new tools for peroxidase family classification. Nucleic Acids Research 37 supp/1: D261-D266.
5. Skamnioti P, Henderson C, Zhang Z, Robinson Z, Gurr SJ (2007) A Novel Role for Catalase B in the Maintenance of Fungal Cell-Wall Integrity During Host Invasion in the Rice Blast Fungus Magnaporthe grisea. Molecular Plant-Microbe Interactions 20: 568-580.
6. Zamocky M, Furtmuller PG, Obinger C (2009) Two distinct groups of fungal catalase/peroxidases. Biochemical Society Transactions 37: 772-777.
7. Chi MH, Park SY, Kim S, Lee YH (2009) A Novel Pathogenicity Gene Is Required in the Rice Blast Fungus to Suppress the Basal Defenses of the Host. Plos Pathogens 5: -.
8. Tanabe S, Ishii-Minami N, Saitoh KI, Otake Y, Kaku H, et al. (2011) The Role of Catalase-Peroxidase Secreted by Magnaporthe oryzae During Early Infection of Rice Cells. Molecular Plant Microbe Interactions 24: 163-171.
9. Bohnert HU, Fudal I, Dioh W, Tharreau D, Notteghem J-L, et al. (2004) A Putative Polyketide Synthase/Peptide Synthetase from *Magnaporthe grisea* Signals Pathogen Attack to Resistant Rice. Plant Cell 16: 2499-2513.
10. Collemare J, Billard A, Boehnert HU, Lebrun MH (2008) Biosynthesis of secondary metabolites in the rice blast fungus *Magnaporthe grisea*: the role of hybrid PKS-NRPS in pathogenicity. Mycological Research 112: 207-215.
11. Park J, Park J, Jang S, Kim S, Kong S, et al. (2008) FTFD: an informatics pipeline supporting phylogenomic analysis of fungal transcription factors. Bioinformatics 24: 1024-1025.
12. Kershaw MJ, Talbot NJ (2009) Genome-wide functional analysis reveals that infection-associated fungal autophagy is necessary for rice blast disease. Proceedings of the National Academy of Sciences of the United States of America 106: 15967-15972.
13. Liu TB, Chen GQ, Min H, Lin FC (2009) MoFLP1, encoding a novel fungal fasciclin-like protein, is involved in conidiation and pathogenicity in *Magnaporthe oryzae*. Journal of Zhejiang University-Science B 10: 434-444.
