# Supplementary material for: Fungal Virulence and Development Is Regulated by Alternative Pre-mRNA 3′End Processing in Magnaporthe oryzae
Source: PLoS Pathog. 2011 Dec 15;7(12):e1002441. doi: 10.1371/journal.ppat.1002441 (PMC3240610; doi:10.1371/journal.ppat.1002441)
Supplement: Table S2 — List of characterised RRM/RGG-containing proteins. (PDF) [file ppat.1002441.s007.pdf]

Table S2. List of characterised RRM/RGG-containing proteins.

Organism	ID	Protein	Domains	aa	Functions	Binding	Complexes	Localisation	References
**Human**	P38159	hnRNP G	1xRRM / 2xRGG	391	Splicing - export		spliceosome C		[1]
	Q86V81	THOC4	1xRRM / 2xRGG	257	Export-decay	proteins and RNA	THO complex		[2]
	Q14011	CIRPB	1xRRM / 3xRGG	172	Suppression of cell proliferation				[3]
	P35637	FUS	1xRRM / 19xRGG	526	Annealing complementary ssDNA	dsDNA / ssDNA		nucleus (Nu)	[4]
	Q01844	EWS	1xRRM / 22xRGG	656	Transcription	RNA		cell membrane, Nu/cytoplasm	[5]
	Q92804	RB56	1xRRM / 22xRGG	592	Transcription	RNA / ssDNA (TATA)	RNA Pol II		[4]
	Q13283	G3BP	1xRRM / 2xRGG	466	Formation stress granules				[6]
	P09651	hnRNP A1	2xRRM / 3xRGG	372	Splicing - export, telomere biogenesis	d(TTAGGG)n	spliceosome C	Nu/cytoplasm	[7,8,9]
	P22626	hnRNP A2/B1	2XRRM / 4xRGG	353	Splicing-localisation telomere maintenance		spliceosome C	Nu/cytoplasm	[10,11]
	P51991	hnRNP A3	2xRRM / 5xRGG	378	Splicing-A2RE-containing mRNA export	cis-acting A2 RE	spliceosome C	Nu/cytoplasm	[1]
	Q14103	D (AUF1) 4 isoforms	2xRRM / 3xRGG	355	mRNA stability / turnover; Recombination	AREs-3'UTR; d(TTAGGG)n; d(CCCTAA)n			[12]
	P23246	SFPQ	2xRRM / 3xRGG	707	Splicing-nuclear retention defective RNAs	intronic polypyrimidine tracts			[13]
	Q13151	hnRNP A0	2xRRM / 3xRGG	305	Splicing		spliceosome C		[1]
	O60506	hnRNP Q 3 isoforms	3xRRM / 8xRGG	623	Splicing	poly(A) / poly(U) RNA			[14]
	O43390	hnRNP R	3xRRM / 8xRGG	633	splicing		spliceosome C		[1]
	P19338	nucleolin	4xRRM / 9xRGG	710	cell surface receptor	cytokines MK and HB-19		cell membrane, Nu/cytoplasm	[15]
**Yeast**	Q01560	Npl3/Nop3	2xRRM / 15xRGG	414	Splicing-export; pre-rRNA processing; transcription, translation			Nu/cytoplasm	[16,17,18,19,20,21]
	Q99383	Hrp1/Nab4	2xRRM / 3xRGG	534	Polyadenylation, chromatin assembly	polyA signal sequences	CF I	Nu/cytoplasm	[22,23]
**Fly**	AAF54963	Squid/hrp4 4 isoforms	2xRRM / 8xRGG	344	mRNA localisation				[24]

References (Table S2)

1. Jurica MS, Licklider LJ, Gygi SP, Grigorieff N, Moore MJ (2002) Purification and characterization of native spliceosomes suitable for three-dimensional structural analysis. RNA 8: 426-439.
2. Strasser K, Masuda S, Mason P, Pfannstiel J, Oppizzi M, et al. (2002) TREX is a conserved complex coupling transcription with messenger RNA export. Nature 417: 304-308.
3. Nishiyama H, Higashitsuji H, Yokoi H, Itoh K, Danno S, et al. (1997) Cloning and characterization of human CIRP (cold-inducible RNA-binding protein) cDNA and chromosomal assignment of the gene. Gene 204: 115-120.
4. Morohoshi F, Arai K, Takahashi E, Tanigami A, Ohki M (1996) Cloning and mapping of a human RBP56 gene encoding a putative RNA binding protein similar to FUS/TLS and EWS proteins. Genomics 38: 51-57.
5. Rabbitts TH, Forster A, Larson R, Nathan P (1993) Fusion of the Dominant-Negative Transcription Regulator Chop with a Novel Gene Fus by Translocation T(12-16) in Malignant Liposarcoma. Nature Genetics 4: 175-180.
6. Tourriere H, Chebli K, Zekri L, Courselaud B, Blanchard JM, et al. (2003) The RasGAP-associated endoribonuclease G3BP assembles stress granules. Journal of Cell Biology 160: 823-831.
7. Pollard AJ, Krainer AR, Robson SC, Europe-Finner GN (2002) Alternative splicing of the adenylyl cyclase stimulatory G-protein G alpha(s) is regulated by SF2/ASF and heterogeneous nuclear ribonucleoprotein A1 (hnRNPA1) and involves the use of an unusual TG 3 '-splice site. Journal of Biological Chemistry 277: 15241-15251.
8. Izaurralde E, Jarmolowski A, Beisel C, Mattaj IW, Dreyfuss G, et al. (1997) A role for the M9 transport signal of hnRNP A1 in mRNA nuclear export. Journal of Cell Biology 137: 27-35.
9. Fisette JF, Toutant J, Dugre-Brisson S, Desgroseillers L, Chabot B (2010) hnRNP A1 and hnRNP H can collaborate to modulate 5 ' splice site selection. RNA 16: 228-238.
10. Hoek KS, Kidd GJ, Carson JH, Smith R (1998) hnRNP A2 selectively binds the cytoplasmic transport sequence of myelin basic protein mRNA. Biochemistry 37: 7021-7029.
11. Moran-Jones K, Wayman L, Kennedy DD, Reddel RR, Sara S, et al. (2005) hnRNP A2, a potential ssDNA/RNA molecular adapter at the telomere. Nucleic Acids Research 33: 486-496.
12. Kajita Y, Nakayama J, Aizawa M, Ishikawa F (1995) The UUAG-Specific RNA-Binding Protein, Heterogeneous Nuclear Ribonucleoprotein D0 - Common Modular Structure and Binding Properties of the 2xRBD-Gly Family. Journal of Biological Chemistry 270: 22167-22175.
13. Patton JG, Porro EB, Galceran J, Tempst P, Nadalginard B (1993) Cloning and Characterization of Psf, a Novel Premessenger Rna Splicing Factor. Genes & Development 7: 393-406.
14. Mourelatos Z, Abel L, Yong JS, Kataoka N, Dreyfuss G (2001) SMN interacts with a novel family of hnRNP and spliceosomal proteins. Embo Journal 20: 5443-5452.
15. Said EA, Krust B, Nisole S, Svab J, Briand JP, et al. (2002) The anti-HIV cytokine midkine binds the cell surface-expressed nucleolin as a low affinity receptor. Journal of Biological Chemistry 277: 37492-37502.
16. Gilbert W, Siebel CW, Guthrie C (2001) Phosphorylation by Sky1p promotes Npl3p shuttling and mRNA dissociation. RNA 7: 302-313.
17. Lee MS, Henry M, Silver PA (1996) A protein that shuttles between the nucleus and the cytoplasm is an important mediator of RNA export. Genes & Development 10: 1233-1246.
18. Kress TL, Krogan NJ, Guthrie C (2008) A Single SR-like Protein, Npl3, Promotes Pre-mRNA Splicing in Budding Yeast. Molecular Cell 32: 727-734.
19. Russell ID, Tollervey D (1992) Nop3 Is an Essential Yeast Protein Which Is Required for Pre-Ribosomal RNA Processing. Journal of Cell Biology 119: 737-747.

20. Dermody JL, Dreyfuss JM, Villen J, Ogundipe B, Gygi SP, et al. (2008) Unphosphorylated SR-Like Protein Npl3 Stimulates RNA Polymerase II Elongation. PLOS One 3: -.
21. Windgassen M, Sturm D, Cajigas IJ, Gonzalez CI, Seedorf M, et al. (2004) Yeast shuttling SR proteins Npl3p, Gbp2p, and Hrb1p are part of the translating mRNPs, and Npl3p can function as a translational repressor. Molecular and Cellular Biology 24: 10479-10491.
22. Kessler MM, Henry MF, Shen E, Zhao J, Gross S, et al. (1997) Hrp1, a sequence-specific RNA-binding protein that shuttles between the nucleus and the cytoplasm, is required for mRNA 3'-end formation in yeast. Genes & Development 11: 2545-2556.
23. Guisbert KSK, Li H, Guthrie C (2007) Alternative 3 ' pre-mRNA processing in Saccharomyces cerevisiae is modulated by Nab4/Hrp1 in vivo. Plos Biology 5: 15-22.
24. Matunis EL, Kelley R, Dreyfuss G (1994) Essential Role for a Heterogeneous Nuclear Ribonucleoprotein (Hnrnp) in Oogenesis - Hrp40 Is Absent from the Germ-Line in the Dorsoventral Mutant Squid. Proceedings of the National Academy of Sciences of the United States of America 91: 2781-2784.
