# Supplementary material for: Fungal Virulence and Development Is Regulated by Alternative Pre-mRNA 3′End Processing in Magnaporthe oryzae
Source: PLoS Pathog. 2011 Dec 15;7(12):e1002441. doi: 10.1371/journal.ppat.1002441 (PMC3240610; doi:10.1371/journal.ppat.1002441)
Supplement: Table S3 — Primers used in this study. (PDF) [file ppat.1002441.s008.pdf]

Table S3. Primers used in this study

Primer name	Sequence 5' → 3'
**General primers**	
2SKF-KpnI	AAAGGTACCAGGGAATAAGGGCGACACGGA
2SKR-KpnI	TATGGTACCTCGCCCTTCCCAACAGTTGCG
M13F	CGCCAGGGTTTTCCCAGTCACGAC
M13R	AGCGGATAACAATTTCACACAGGA
T3	AAATTAACCCTCACTAAAGGGA
AT-RB	GATTGTCGTTTCCCGCCTTCAG
AT-LB2	CCAGTACTAAAATCCAGATCCC
34568-PstI	GCCTGTCGCGTCCTACTGC
30779-PstI	GCACAGAGGTGGCGCTGAGGC
**Primers for RBP35 expression in *E. coli***	
Forward NdeI	TTCATATGGGTCATCATCATCATCATCACGCTGGTGCCGAGGAGGATTTC
Reverse XhoI	TTCTCGAGTGTCAGATATCCATTTGCTATTCAGGTCGAGGACGTTTGGCACC
**Primers used for Southern and Northern blot hybridisation with *RBP5* probe**	
M35-GSP1	CCACGTCCGCGGTTGTTATAACCTCCG
M35-GSP2	CGTCTCCACCCTGCTGCTCCTGG
5-M35RGG	CGGCGGAGGTGGCAATTTC
M35-RT2	GTCCTGAATTCGCCATTAGATCAGCTTTCAG
**Primers for construction of RBP35-mCherry variants**	
B3-35	GGGGACAACTTTGTATAATAAAGTTG-GAAGACAAGATAAGATGAGTTGCC
B1RC-35	GGGGACTGCTTTTTTGTACAAACTTG-ATTCCGGGCGAGGACGTTTGGCAC
B1RNt-35	GGGGACTGCTTTTTTGTACAAACTTG-TGTGTGAAAATTTGCGAGCTTGATGC
B4-RBP35	GGGGACAACTTTGTATAGAAAAGTTG-GCCTTCTCATTGGTGGTTAGCCACC
**Primers for 5' and 3' RACE cloning of *RBP35* cDNA**	
GSP1a	CCACGTCCGCGGTTGTTATAACCTCCG
GSP2a	GCTGTCAAGCATCGCCTGGAATCCTCAG
NGSP2	CGAGGATGGCGCTTCACAAGC
**Primers for construction of Nop1-mCherry fusion constructs**	
B4-Nop1	GGGGACAACTTTGTATAGAAAAGTTG-GAGTAGCCACGGAGTTCATCCAC
B3-Nop1	GGGGACAACTTTGTATAATAAAGTTG-TGTTGAATCCATACTGCTATGTCC
B1RC-Nop1	GGGGACTGCTTTTTTGTACAAACTTG-ATTCGATAAATTCCCTCTGCTGGT
B2RC-Nop1	GGGGACAGCTTTCTTGTACAAAGTGG-GATTGTGACGAATGGCTGTGAAAG
**Primers used in qPCR experiments**	
ZnTrFc fw2	ACGAGATCCTCAAGCAGCAT
ZnTrFc Rv2	TTAACATTCCCGCTGACCTC
MRS2-splicing fw1	CCACAGCAAGAATGGGATTT
MRS2-splicing rv1	CTTGTAGCTGGTCCCTGAGC
Lacasse fw1	TCTTCCAGTACGAGGGTGCT
Lacasse rv1	GACGGGCGAGAAGTTGATAA
40s fw	TCTACCCCGTCGAGATTGTC
40s rv	TAGGTGTCGAGGCGGTAGTC
eIF3i fw	CCATGACCATCGTCTGTGAC
eIF3i rv	CGTCGTATTGCGAGACTGAG
Ku80 fw	CCCGGAGACATACAAGGAGA
Ku80 rv	CCCTCAAAATCCTGTCCTGA
NirA-Fw	AGCCCAAGGATGGCCAGTTA
NirA-Rv	CATATACGGCGAGGCGACAC
NiaD-Fw	GCAGCGAGGAGTTCCTGAC
NiaD-Rv	AGGACGCGGTTCTGTGGATT
FKBP-4-Fw	TCGACATGGAGGAGCACG
FKBP-4-Rv	AGCCCTCTCACCCTCTCC
Mep2-Fw	CTGGTACGCGAGCTGGATG
Mep2-Rv	CGACATCTTTGCCGAGCAC

Tap42-Fw	ACCTCCCGTTCTTGCTGACC
Tap42-Rv	CTCGTAGCCGTCGAGGAGG
Avo3-Fw	CGCGTTCCAGATCTGCTTTG
Avo3-Rv	TGCCGCCGGTTATAGAATGA
TorA-Fw	TGCACCAACCGAGTTCTGC
TorA-Rv	AGTCTGGCCCACAGCAACTG
TamA-fw1	GCATACTTTGCCACCGAAAT
TamA-rv1	GTACCTGTCGCCAGCTCTTC
actin Fw	CCTGGCACCGTCGTCGATGAAGG
actin Rv	GCGAGGCGAGAATGGAACCAC
TrFc37_CDS-Fw-qPCR	GCAAAAGGAGCTGGCCAAG
TrFc37_UTR-Fw-qPCR	TATGCAGATTGGTTGATCC
Asp_CDS-Rv-qPCR	GAACGCCATCTCGACATCAC
TrFc39_3UTR-Fw-qPCR	TCCCCCATTTATGAACGTTG
TrFc39_CDS-Rv-qPCR	GAAGCCAAAGTCCACAGTCG
**Primers used in RT-PCR experiments**	
14-3-3-Fw-CDS	GCTCTCGGTTGCCTACAAG
14-3-3-Rv1-CDS	TGCATGATAAGGGTGCTGTC
14-3-3-Rv2-UTR	GACGCAAATTACTTGGAGGG
S7-40S-Fw-CDS	ACGACCTCGAGACGAACACTG
S7-40S-Rv1-CDS	TAGGTGTCGAGGCGGTAGTC
S7-40S-Rv2-UTR	GCCGCTTATTTGCAGAGTAG
Asp-Fw-CDS	AAGCAGTCCTCCCCTATGGT
Asp-Rv1-CDS	CTCGCTCTCAAGCTTGTCCT
Asp-Rv2-UTR	CTCGCCGATACCATTTCAGTC
TrFc39-CDS	ATGAGAGAGCAACCGCAACT
TrFc39-Rv1-CDS	GCTGTTGTCAGCACTGGTGT
TrFc39-Rv2-UTR	TTCGTGACATCATGGCGCAG
TrFc37-CDS	GTCGACGAGACCCCAATCT
TrFc37-Rv1-CDS	CTCGGCGTACTTCTCCTCTG
TrFc37-Rv2-UTR	CAGCATACTTCATATCCACAG
